# Peri-transplant eculizumab for preventing antibody-mediated rejection in kidney transplant recipients at high immunologic risk: a systematic review with design-stratified evidence synthesis

**DOI:** 10.3389/fimmu.2026.1982870

**Published:** 2026-10-20

**Authors:** Chengze Liang, Rong Bao, Saifu Yin, Turun Song, Tao Lin

**Affiliations:** 1 Department of Urology/Institute of Urology, West China Hospital, Sichuan University, Chengdu, Sichuan, China; 2 West China Medical School, Sichuan University, Chengdu, Sichuan, China; 3 Kidney Transplantation Center, West China Hospital, Sichuan University, Chengdu, Sichuan, China; 4 Organ Transplantation Center, West China Hospital, Sichuan University, Chengdu, Sichuan, China

**Keywords:** antibody-mediated rejection, complement inhibition, desensitization, eculizumab, kidney transplantation

## Abstract

**Introduction:**

Highly sensitized candidates, preformed donor-specific antibodies, and ABO incompatibility restrict access to kidney transplantation and increase antibody-mediated rejection (AMR) risk. We systematically evaluated peri-transplant eculizumab-containing prophylaxis in high-immunologic-risk kidney transplantation.

**Methods:**

We searched PubMed, Europe PMC, Ovid Embase, Web of Science Core Collection, Ovid CENTRAL, ClinicalTrials.gov, WHO ICTRP, and supplementary sources; database searches ended 10 June 2026, and registry searches ended 20 August 2026.

**Results:**

A total of 31 reports were included; 30 were linked to 14 studies or registry projects, and one pathology report was retained qualitatively. In the only randomized trial, centrally confirmed AMR occurred in 5/51 recipients in both the eculizumab and standard-desensitization groups [risk ratio (RR) 1.00, 95% confidence interval (CI) 0.31–3.25]. A historical comparison reported AMR in 2/26 versus 21/51 recipients (RR 0.19, 95% CI 0.05–0.74), but treatment era, post-transplant plasma exchange, and clinical management changed concurrently. An 80-recipient single-arm study reported three AMR events by week 9. Long-term follow-up showed no consistent graft-survival or kidney-function advantage, while persistent donor-specific antibodies and chronic microvascular injury remained evident.

**Discussion:**

Eculizumab can provide terminal complement blockade within selected combination prophylaxis, but randomized evidence does not establish an incremental reduction in AMR. Definitive trials require standardized background therapy, rescue criteria, long-term graft outcomes, and infection surveillance. This systematic review was registered in PROSPERO (CRD420261484542).

**Systematic review registration:**

https://www.crd.york.ac.uk/PROSPERO/, identifier CRD420261484542.

## 1 Introduction

Highly sensitized and pre-existing donor-specific antibodies (DSA) can narrow the pool of acceptable donors (1–4). Transplantation across DSA or positive crossmatch will increase the risk of antibody-mediated rejection (AMR) after kidney transplantation (5). ABO blood group incompatibility is another type of antibody barrier that can also cause humoral immune damage (6–8). For some candidates, human leukocyte antigen (HLA)-incompatible living donor kidney transplantation has a survival benefit compared with continuing to wait, but it entails a higher risk of AMR (5, 9). Kidney pair exchange, preferential allocation, and acceptable mismatches can circumvent some antibody barriers (1–3). When a compatible kidney donor cannot be obtained, plasma exchange (PLEX) or immunoadsorption is often used in combination with intravenous immunoglobulin (IVIG) and B cell-targeted therapy (2, 10, 11). These regimens can reduce circulating antibodies, but do not consistently prevent DSA rebound (10, 11). Imlifidase can rapidly cleave IgG, but in the Highdes trial, DSA often rebounded 3 to 14 days after surgery, and 38.9% of recipients developed early biopsy-proven AMR (12).

Binding of pre-existing DSA to graft endothelial HLA activates the classical complement pathway. C5 cleavage produces C5a and promotes the assembly of the C5b-9 membrane attack complex, which is involved in inflammatory cell recruitment, endothelial activation, and early graft damage (5, 13). Eculizumab (ECU) blocks C5 cleavage (13, 14). Establishing C5 inhibition before and after reperfusion can theoretically limit early terminal complement-mediated damage (**Figure 1**) (13, 15). ECU does not clear DSA, nor does it block upstream C3 activation, C4d deposition, or complement-independent antibody effects (5, 13, 16). Therefore, C5 blockade only covers one effector link in the combined prophylactic regimen, and its clinical results also depend on antibody removal, IVIG, maintenance immunosuppression, and the timing of administration (13, 15, 16).

**Figure 1:**
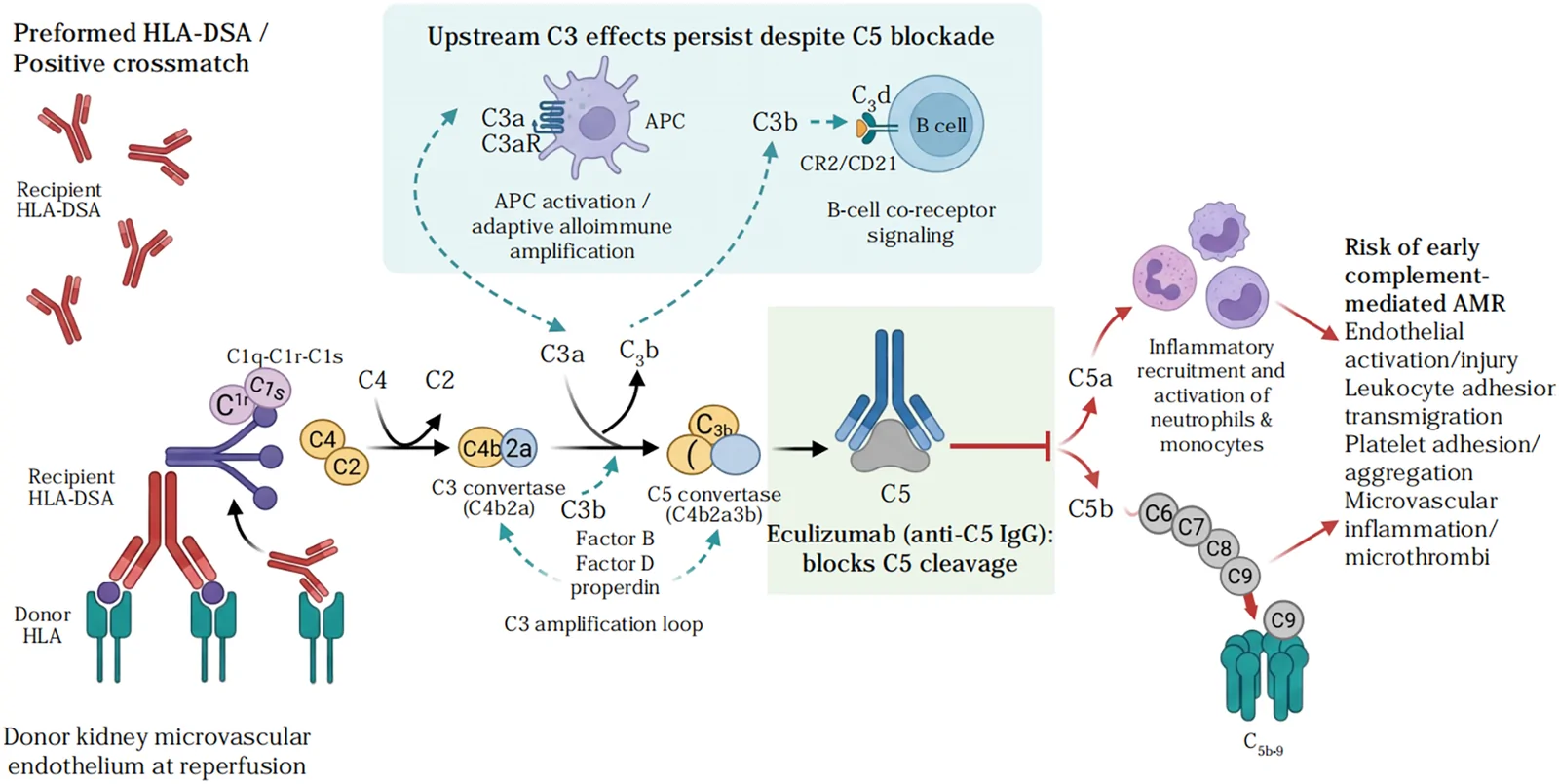
Biological rationale for terminal complement blockade with eculizumab. Pre-existing HLA-DSA binds to donor HLA to initiate the classical complement pathway. Eculizumab binds C5 and prevents its cleavage, thereby reducing C5a-mediated inflammatory cell recruitment and C5b-9 membrane attack complex formation. C5 blockade does not clear DSA, nor does it block upstream C3 activation, C4d deposition, or complement-independent antibody effects; thus, its theoretical role focuses on the early terminal complement-mediated injury window after transplant reperfusion (5, 13, 15, 16). Created in BioRender. Xing, H. (2026). https://BioRender.com/mtkxakn.

The core evidence for ECU prevention of AMR in kidney transplantation comes from randomized trials, historical comparisons, and multicenter single-arm studies (15, 17, 18). Long-term follow-up mainly comes from the Stegall cohort and its extended follow-up (19, 20). ABO incompatibility scenarios are mainly based on small case studies (6–8). Other positive crossmatch studies similarly provide only experience with small sample sizes (21, 22). Part of the evidence also comes from mixed indication cases and conference abstracts (23, 24). Previous reviews usually discussed ECU or complement-directed therapy more broadly in kidney transplantation (25–27). Another review focuses on the treatment of AMR that has occurred (28). The previous reviews identified in this search did not systematically address multiple reports and overlapping populations in the evidence for peri-transplant prevention in ECU by study. Expert consensus and guidelines are also inconsistent with the clinical role of ECU (29, 30). This systematic review integrates AMR, graft and patient outcomes, kidney function, DSA, pathology, and infection safety according to study design and clinical scenarios; evaluates whether ECU-containing prophylactic regimens have a demonstrable incremental clinical benefit; and defines the applicable scope of the evidence.

## 2 Methods

This systematic review was registered in PROSPERO (CRD420261484542) and reported in accordance with PRISMA 2020 (31).

### 2.1 Eligibility criteria

High immunologic risk was defined as HLA sensitization or pre-existing DSA, a positive flow-cytometric or complement-dependent cytotoxicity crossmatch, or ABO incompatibility. Studies were grouped into three clinical settings: HLA-incompatible living-donor transplantation, deceased-donor transplantation in recipients with pre-existing DSA, and ABO-incompatible transplantation.

We included ECU monotherapy or ECU-containing prophylactic regimens initiated before established AMR, without restrictions on dose, timing, treatment duration, or concomitant immunologic therapy. Comparative studies informed the incremental effect of ECU, whereas uncontrolled cohorts and case-based reports were used only to describe implementation, outcomes, and safety. We excluded studies primarily evaluating ECU for established AMR, recurrent atypical hemolytic uremic syndrome or thrombotic microangiopathy, delayed graft function, or ischemia–reperfusion injury. Studies in which AMR prophylaxis could not be separated from an aHUS/TMA indication were retained only for qualitative interpretation.

### 2.2 Information sources and search strategy

We searched PubMed, Europe PMC, Ovid Embase, Web of Science Core Collection, Ovid CENTRAL, ClinicalTrials.gov, and WHO ICTRP, supplemented by OpenAlex, Crossref, Semantic Scholar, conference sources, and citation tracking. The main search was completed on 10 June 2026, and the supplementary WHO ICTRP search was completed on 20 August 2026. Searches combined ECU/Soliris and kidney-transplantation terms without restrictions on language, publication year, age, or study design. AMR and prophylaxis terms were not required to preserve sensitivity. The initial exports from open-access sources comprised 6,805 records. Subsequent imports contributed 1,751 records from Ovid Embase, 685 records from Web of Science Core Collection, 35 records from Ovid CENTRAL, and 15 registry records after the removal of within-search overlap in the supplementary WHO ICTRP searches. These sources contributed 9,291 records before the topical filter and duplicate removals described below. Citation tracking and conference searches identified a further 211 records.

Before human title/abstract screening, a deterministic topical filter was applied only to the initial 6,805-record set. It required an ECU/Soliris term, a kidney/renal term, and a transplant/allograft/graft term in the title, abstract, or indexed metadata; it removed 5,219 records and retained 1,586. Deduplication of these 1,586 records yielded 689 unique records. After deduplication against the existing collection, subsequent imports from Embase, Web of Science, CENTRAL, and WHO ICTRP contributed 1,084, 168, 4, and 1 additional record, respectively. Thus, 1,946 records from databases and registers underwent title/abstract screening. The topical filter was a record-management step and did not determine eligibility. Source-specific search strategies are provided in **Supplementary Table 1**, and the study-selection flow is shown in **Figure 2**.

**Figure 2:**
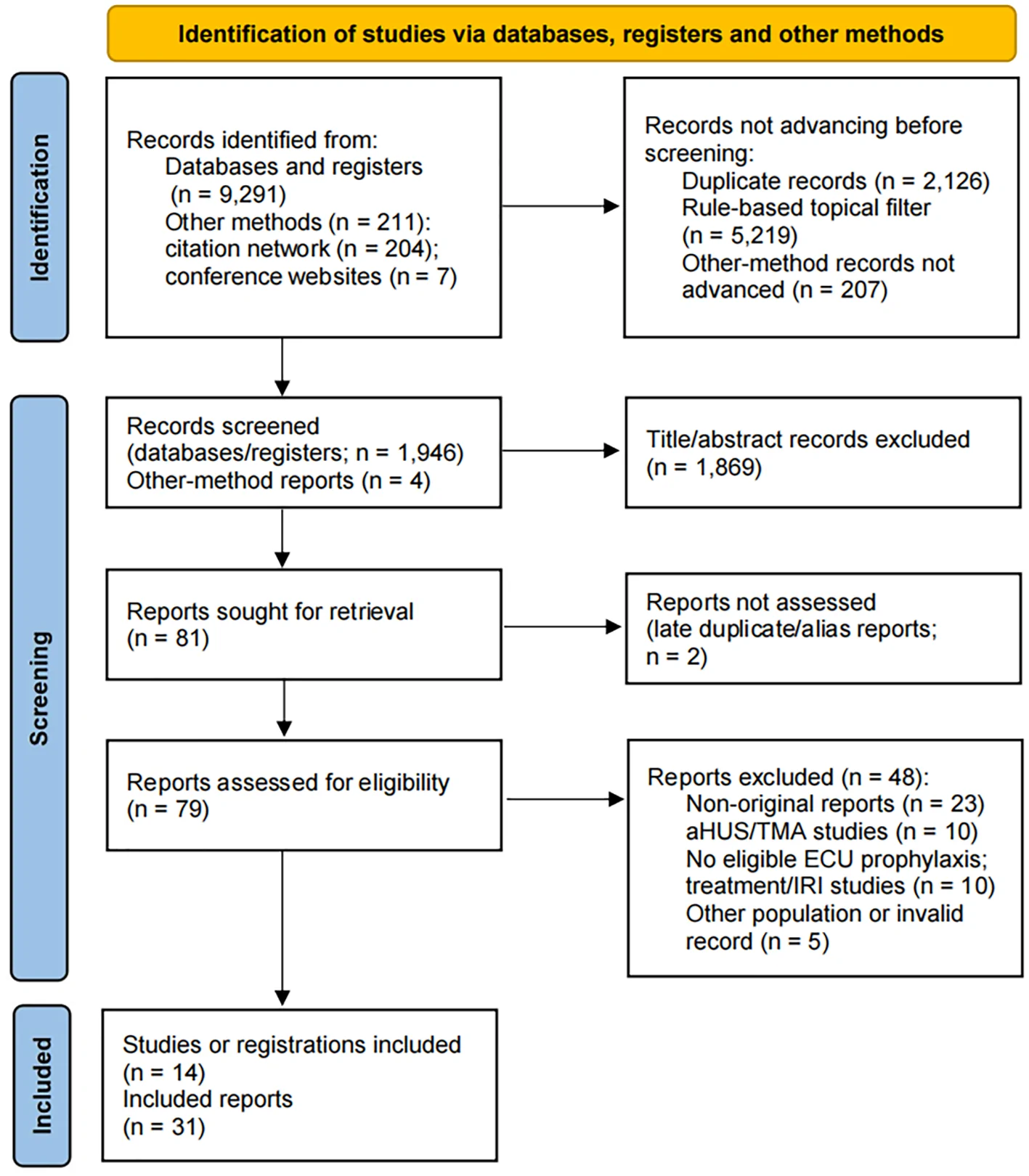
Study selection flow diagram. PRISMA 2020 flowchart. The figure shows records obtained from database searches, registration platforms, and other sources, respectively. In the full-text stage, 81 records were processed, 2 late-identified duplicate records were deleted and 79 unique reports were evaluated; 48 were excluded and 31 were included. Among them, 30 were classified into 14 studies or registry projects, and the other pathology companion report with an unknown source of the cohort was only used as a qualitative supplement. The reasons for exclusion of the full text are shown in **Supplementary Table 2**.

### 2.3 Study selection and management of multiple reports

After deduplication, Chengze Liang and Rong Bao independently screened all titles and abstracts and assessed potentially eligible full-text reports against the prespecified eligibility criteria. The same reviewers independently performed data extraction and risk-of-bias assessments. Disagreements were resolved through discussion and consensus; unresolved disagreements were adjudicated by Saifu Yin. Saifu Yin also retrieved full texts, verified duplicate and alias records, and assisted with source location. No automation tools made eligibility decisions; the topical filter described above was limited to record management before human screening.

Multiple reports were linked using study center, enrollment period, sample size, intervention protocol, and authorship. The unit of analysis was the study or cohort rather than the individual report. Peer-reviewed primary reports took precedence over conference abstracts and companion analyses. When conflicting definitions, denominators, or values could not be resolved, they were retained separately rather than combined. Report linkage, cohort overlap, and source selection are detailed in **Supplementary Table 3**. Additional companion publications and conference reports were linked to their corresponding study projects rather than counted as independent studies (46–54).

### 2.4 Data extraction and outcome definitions

Data were extracted by study, treatment arm, outcome, time point, and reporting source. Extraction records were compared, and discrepancies were resolved against the original article, registry record, or conference report. Unreported values were not treated as zero, Kaplan–Meier estimates were not converted to event counts, and unresolved source-specific denominators were retained.

AMR outcomes were classified as centrally assessed biopsy-confirmed clinical AMR, locally assessed AMR, subclinical AMR, study-defined composite treatment failure, investigator-judged rescue treatment, or individual histologic components such as C4d deposition and microvascular inflammation. These outcomes were not substituted for one another or combined into a single event rate. We used AMR throughout; when an original report used ABMR or a specific Banff classification, the original terminology and definition were retained.

Secondary outcomes included graft loss or survival, death, kidney function, DSA, C4d deposition, microvascular inflammation, transplant glomerulopathy, serious adverse events, and infection. ECU dose and timing, treatment duration, induction and maintenance immunosuppression, IVIG, and PLEX were also extracted. PLEX was recorded by timing, frequency, planned or rescue use, and technique but was not treated as an independent efficacy outcome.

### 2.5 Risk-of-bias assessment

RoB 2 was used for randomized trials, ROBINS-I V2 (November 2025 draft) was used for non-randomized comparative studies, and the applicable JBI Critical Appraisal Checklists were used for single-arm cohorts, case series, and case reports.

Composite treatment failure and centrally assessed pathologic AMR in the Marks trial were evaluated separately. Prespecified confounding domains for the Stegall historical comparison included treatment era, antibody-removal strategy, risk selection, biopsy strategy, rescue therapy, and follow-up. Detailed judgments, supporting rationale, funding, and conflicts of interest are reported in **Supplementary Table 4**. Comparative findings were interpreted according to study design, risk of bias, precision, clinical directness, outcome definitions, and co-interventions. No formal certainty-of-evidence framework was applied.

### 2.6 Evidence synthesis and statistical analysis

The synthesis followed the SWiM reporting guideline (32). Evidence was organized by randomized, historical-control, other non-randomized, single-arm, and case-based designs and then stratified by the three transplant settings. Follow-up was classified as early, principally 9 weeks or 3 months; intermediate, 6–12 months; or long term, more than 12 months. Effect direction was interpreted only when the outcome definition and comparison context were adequate.

Risk ratios (RRs), risk differences, and 95% confidence intervals (CIs) were calculated when 2 × 2 tables were available. RRs used Wald CIs on the log scale. When one or more cells contained zero events, 0.5 was added to all four cells; RRs were not calculated for double-zero comparisons. Risk-difference CIs used the Newcombe hybrid-score method without continuity correction, and single-arm proportions used Clopper–Pearson exact CIs.

Randomized and historical-control evidence were not pooled. Meta-analysis, heterogeneity statistics, funnel plots, meta-regression, and subgroup analyses were not undertaken because the studies were not sufficiently exchangeable in design, population, co-interventions, AMR definitions, biopsy strategies, or follow-up.

## 3 Results

### 3.1 Study selection and characteristics

Databases and registers contributed 9,291 imported records, while citation-network and conference searches contributed 211 records. After the staged topical filtering and deduplication described in Methods, 1,946 records from databases and registers underwent title/abstract screening. Of these, 1,869 were excluded, leaving 77 reports for retrieval. Four additional reports identified through other methods brought the total sought for retrieval to 81. Two late-identified duplicate or alias reports were not assessed, leaving 79 unique reports for full-text evaluation. Forty-eight were excluded (**Supplementary Table 2**), and 31 met the inclusion criteria (**Figure 2**). Thirty of these were linked to 14 independent studies or registered projects. The source cohort of the Brealey 2018 pathology companion report cannot be confirmed. It was retained only as qualitative evidence and is not included in the number of projects or patients (33). Among the 14 studies or registry projects, 12 have reported clinical results and 2 only have registry protocols (34, 35). **Table 1** uses cohorts or registry protocols as row units, with a total of 15 rows; the number of rows is greater than the number of projects because the Chicago project includes both West-Thielke/Campara’s four-case cohort and Heagler’s nine-case cohort. The overlap between the two is uncertain, so they are kept in separate rows within the same project without adding up (6, 21, 37). NCT06453135 and ChiCTR2500114806 have different centers, sponsors, dosages, and enrollment protocols, and they belong to two independent registry projects without posted results (34, 35).

**Table 1:** Design, population, and clinical setting of included studies.

Research, region and design	Sample, age and gender	Donor and recipient background	Baseline immunologic risk	AMR definition and biopsy strategy	ECU and primary co-intervention	Follow-up	References
Marks 2019; 41 centers in 10 countries; open-label randomized phase 2 trial	104 cases were randomized; 102 cases were actually transplanted and included in the published analysis, 51/group; group NR of two non-transplanters; ECU group 45.0±14.5 years old, 37/51 women; control group 40.9±13.2 years old, 30/51 women	Living donor kidney transplant requiring desensitization; 24/51 and 35/51 previous transplants; 46/51 in each group received dialysis	DSA positive 49/51 and 47/51; immunodominant DSA average MFI 8135 and 8741; CDC positive 36/51 and 28/51 during the screening period, all of which can have positive flow cross-matching	Central pathology Banff 2007 Grade II/III AMR; Indicated biopsy and post-reperfusion, day 14, 3/12/36 month protocol biopsy	Both groups shared pretransplant PLEX±IVIG as defined by each center; ECU group received a 9-week multi-dose regimen; the same induction and maintenance immunosuppression	Primary endpoint 9 weeks; average participation approximately 27–29 months, maximum 36 months	(17)
Glotz 2019; 15 centers in Europe and Australia; open-label single-arm phase 2 trial	80 cases; median age 52 (24–70); female 48/80	High-risk deceased donor kidney transplantation; 80/80 dialysis, average dialysis period 162.2 months	CDC negative at the time of transplantation; previous CDC positivity, B/T cell flow crossmatch 300–500 mcs or single DSA MFI>3000 are allowed; 71/80 have baseline DSA data	Central pathology Banff 2007 Grade II/III AMR; protocol and indicated biopsies at reperfusion, day 14, 3/12 months	9-week multi-dose ECU; routine pretransplant PLEX/IVIG not scheduled; rescue PLEX, IVIG, and supplemental ECU allowed after AMR	Primary endpoint 9 weeks; follow-up 12 and 36 months	(18)
Stegall 2011 and extended follow-up; US single center; prospective cohort and historical controls	Main comparison 26/51; ECU group 48.6±12.5 years old, 21/26 women; historical control 48.4±11.5 years old, 40/51 women; expansion cohort 30/48	Positive cross-match living donor kidney transplant; previous transplants 13/26 and 13/51	All had pre-existing DSA and positive B-cell flow-cytometric crossmatch; average BFXM 330 and 327 mcs; DSA average MFI 7188 and 6473	Early studies used deterioration of graft function combined with Banff pathology; protocol biopsy on days 7/14/28/90/365 in the ECU group, and follow-up reports were extended to long-term protocol biopsy	Risk-adaptive ECU; 18/26 pretransplant PLEX; routine post-transplant prophylactic PLEX canceled during ECU phase; historical control continued post-transplant PLEX	3-month primary comparison; extended follow-up 2–7 years	(15, 19, 20, 36)
West-Thielke 2018; US single center; case series	4 cases; 48, 71, 55 and 60 years old; 3/4 female	ABO-incompatible living donor kidney transplant; dialysis 23–1408 days	Preoperative IgG titer 1:16–1:2048, pretransplant 1:2–1:32; peak PRA 0%–52%; baseline DSA mostly NR	AMR definition and Banff version NR; biopsy time points are individualized, only 1 case completed the 3/6/12-month series	Preoperative TPEX 3–10 times; multiple doses of ECU over 9 weeks; no post-transplant TPEX; no IVIG or rituximab reported	175–498 days; only 1 case reached 1 year at the time of the main report	(6, 37)
Heagler 2022; US single center; conference case series	9 cases; median age 39 years (23–66); 4/9 women	ABO-incompatible living donor kidney transplantation	ABO titers 1:4–1:512; most PRAs were <10%, and one case had class I/II PRAs both 59%; most DSAs were negative or not tested	1 case of biopsy-confirmed AMR; Banff version and systematic protocol biopsy strategy NR	Single pretransplant ECU 900 mg; no prophylactic PLEX; rescue PLEX after AMR	Figure shows follow-up to 6 months, individual follow-up range NR	(21)
Heo 2024; Korean single center; double case study	2 cases; 67-year-old male, 48-year-old female	ABO incompatible living donor kidney transplantation; both cases were diabetic ESKD	The antibody titers were IgM/IgG 1:512/1:512 and 1:256/>1:1024 respectively; the second case had B54/A33 DSA and T/B-cell flow-cytometric crossmatch was positive.	Banff 2022 review; case-specific indications and follow-up biopsy, results labeled as probable AMR	Add peri-transplant ECU after rituximab, IVIG and intensive PLEX; continue rescue according to clinical conditions after surgery	12 months	(7)
Mitomo 2025; Japanese single center; two-case study	2 cases; 54-year-old female, 70-year-old male	ABO-incompatible living donor kidney transplant; hemodialysis at 2 and 7 years	The anti-A/B titers on admission were 1:2048 and 1:32768, and the average at the time of transplantation was 1:256; the first case had DSA MFI 6321 and T/B-cell flow-cytometric crossmatch was strongly positive, and the second case was negative.	Reperfusion and 3-month protocol biopsy, the first case also has a 1-year biopsy; complete AMR determination rules NR	Preoperative PLEX, rituximab, and high-dose IVIG; single ECU 900 mg before reperfusion	about 1 year	(8)
Yoshikawa 2025; US single center; conference case series	3 cases; age and gender NR	Positive flow cross-match kidney transplant; 2/3 had previous kidney transplant	All 3 cases were positive for T/B-cell flow-cytometric crossmatch and had persistent DSA; the accurate baseline MFI is only shown in the figure, text NR	Accessible abstracts do not report AMR definition or biopsy strategy	2 doses of ECU combined with belatacept-based individualized desensitization	Kidney function 1 month; infection follow-up 6 months	(22)
Okumi 2026; single center in Japan; mixed indication case report	1 case; 41-year-old female	Positive T cell flow cross-matched living donor kidney transplantation with suspected aHUS; converted to hemodialysis after peritoneal dialysis	HLA-B*04:06 DSA MFI 15884; strong positive T/B-cell flow-cytometric crossmatch; anti-A IgG 1:4	Reperfusion biopsy and 3-month, 1-year protocol biopsy; Banff version NR	Preoperative ECU combines PLEX, IVIG and rituximab, and is adjusted according to complement activity; ECU is used for both AMR prevention and suspected aHUS treatment.	1 year	(23)
Shimmura 2017; Japan single center; conference case report	1 case; 29-year-old female	ABO-incompatible living donor kidney transplantation	Baseline anti-A IgG 1:4096, IgM 1:512; HLA-DSA and crossmatch NR	Day 14 and 6 month biopsies; 6 month for biopsy-proven AMR, Banff version NR	DFPP, PLEX, IVIG and 8 doses of ECU	More than 6 months, last follow-up NR	(24)
NCT01106027; US single center; open label registry results cohort	2 cases were treated and 1 case completed the primary endpoint; 18-65 years old, 1/2 female	Positive cross-match deceased donor kidney transplantation; 2 cases both had ESRD	Requires DSA and T or B cell crossmatch >200 at transplant; baseline MFI NR	Banff 2005 Acute Humoral Rejection Criteria; Biopsy Triggers and Protocol Timepoints NR	ECU open label protocol; see registration record for complete co-interventions	1 year	(38)
Wan 2026; Single center in China; Comparative study with unknown grouping method	30/group; ECU+DFPP group 46.4±9.3 years old, 12/30 women; control group 45.2±9.8 years old, 14/30 women	ABO-incompatible kidney transplant; mean dialysis 6.4 and 6.2 months	ABO donor-recipient matching, DSA, PRA and cross-matching are not reported; some table denominators are inconsistent with the text	Only rejection is reported, no definition of AMR or biopsy strategy provided	ECU combined with DFPP versus conventional care; reporting of co-intervention is incomplete	3 months	(39)
Milan 2022/2023; single center in Italy; conference case series and historical controls	7 cases expanded to 10 cases, 20 historical controls; age and gender NR	Highly sensitized deceased-donor kidney transplantation	Experimental group PRA>95%, C1q combined with DSA MFI>3000; historical control arm-specific baseline distribution NR	Report AMR and 6-month protocol biopsy, but Banff version, full definition, and biopsy denominator NR	ECU combined with obinutuzumab multimodal induction; historical control used rituximab regimen	Median 13–16 months, extended reporting range 6–24 months	(40, 41)
NCT06453135; single center in China; registered single-arm protocol	No enrollment results yet; planned adult	ABO-incompatible living donor kidney transplantation	Plan to determine enrollment and ECU dosing by blood group antibody titers; observed baseline data not yet available	Registry records do not provide AMR definition or biopsy strategy	Planned single ECU, no preventive PLEX strategy	Planned primary endpoint 6 months	(34)
ChiCTR2500114806; single center in China; registered single-arm protocol	No enrollment results yet; planned for ≥18 years old	The first ABO-incompatible living donor kidney transplant	Preoperative blood group antibody titer ≥1:64; other observed baseline data not available	Registration records do not provide Banff/clinicopathological definitions or biopsy strategies	Planning a Peritransplant ECU Prevention Program	Planning time point in accessible registration record NR	(35)

ECU, eculizumab; PLEX, plasma exchange; IVIG, intravenous immunoglobulin; DSA, donor-specific antibody; MFI, mean fluorescence intensity; CDC, complement-dependent cytotoxicity crossmatch; BFXM, B-cell flow-cytometric crossmatch; NR, not reported in the original report. Table row units are cohorts or enrollment plans, not individual projects. 14 items correspond to 15 rows because the overlap of 4 patients in the West-Thielke/Campara cohort and 9 patients in the Heagler cohort within the Chicago project cannot be confirmed, and the sample sizes of the two cohorts are not totaled. NCT06453135 and ChiCTR2500114806 are two different registry projects without posted results. Recipients in Stegall's primary cohort and extended follow-up were not double-counted. The Marks and Glotz trials were funded by Alexion Pharmaceuticals; the Stegall report disclosed that Alexion was the study sponsor and that the lead author had a research contract with it (15, 17, 18). The funding and conflicts of interest of each study are shown in **Supplementary Table 4**.

Among the 31 reports, 22 provided patient outcomes, pathology findings, or safety information; 7 were used to check design, reporting chain, or cohort overlap; and 2 only described registry protocols without posted results. Comparative evidence includes the Marks randomized trial (17) and the Stegall historical comparison and its extended follow-up (15, 19, 20). Wan and Milan also included comparator groups but had major methodological limitations (39–41). Glotz is a single-arm study of deceased-donor kidney transplantation (18). The remaining clinical evidence mainly comes from ABO incompatibility case series, case reports, and conference abstracts (6–8, 21–24, 38). Journal articles, long-term follow-up, and companion reports of the same cohort are combined at the study level. Lefaucheur’s *post-hoc* analysis and Dean’s companion reports are not counted as independent studies or independent patients (16, 36). Since some overlap of recipients reported from the same center cannot be completely excluded, this review does not calculate the total number of independent patients across projects (**Supplementary Table 3**).

Mean or median age in the three principal studies ranged from approximately 41 to 52 years, but the immune risks and outcomes were determined differently. Most recipients in the Marks trial had DSA and were randomized to ECU in addition to pre-transplantation therapy as defined by each center (17). The Stegall cohort included living donor kidney transplant recipients with preexisting DSA and positive B-cell flow-cytometric crossmatch (15). The Glotz study included deceased-donor kidney transplant recipients with preexisting DSA, requiring a negative complement-dependent cytotoxicity crossmatch at the time of transplantation (18). The West-Thielke, Heo, and Mitomo studies focused on ABO-incompatible living donor transplantation, and Heagler and Yoshikawa used single-dose or short-course ECU regimens (6–8, 21, 22). All studies added ECU to combined prophylactic regimens including antibody removal, IVIG, induction therapy, or maintenance immunosuppression, with different combinations (**Tables 1**, **2**). The Marks and Glotz trials were funded by Alexion Pharmaceuticals; the Stegall report disclosed that Alexion was the study sponsor and that the lead author had a research contract with the company (15, 17, 18).

**Table 2:** Eculizumab administration and concomitant immunoprophylaxis.

Study	Eculizumab dosage and timing	Pretransplant PLEX or antibody removal	Planned and rescue post-transplant PLEX	Other major co-interventions	Role of ECU in the regimen	References
Marks 2019	1200 mg approximately 1 hour before reperfusion; 900 mg on days 1, 7, 14, 21, and 28 after surgery; 1200 mg on weeks 5, 7, and 9	Both groups received PP+IVIG (93.1%) or PP alone (6.9%) as defined by each center; the number of patients after grouping was NR	Routine post-transplant prophylactic PP is not prescribed in the ECU group; after diagnosis of AMR, both groups can use PP and/or IVIG, and ECU 600 mg is supplemented after PP; in the control group, 14/51 in the first 9 weeks and 22/51 in the full follow-up received ECU due to AMR treatment.	ATG induction; tacrolimus, MMF, and glucocorticoid maintenance	Random ECU incremental treatment based on background treatment in each center	(17)
Glotz 2019	Same 9-week program as Marks	No routine pretransplant PP or IVIG is scheduled	No PP was scheduled in the first 9 weeks, PP/IVIG and post-PP ECU 600 mg were allowed after biopsy confirmed AMR; 59/80 did not receive PP at 12 months	Recommended total ATG dose is 6 mg/kg; tacrolimus, MMF, and glucocorticoid maintenance	Single-arm regimen to avoid planned PP/IVIG in deceased donor kidney transplantation scenario	(18)
Stegall 2011 and extended follow-up	1200 mg before transplantation; 600 mg every week on the 1st day after surgery and the first 4 weeks; if BFXM persists ≥200, 1200 mg every 2 weeks starting from the 5th week and stop according to BFXM	18/26 received pretransplant PE, with an average of 4.0±3.6 times; historical controls 35/51, with an average of 3.7±3.4 times	3/26 in the ECU group and 39/51 in the historical control group received any post-transplant PE; the original report did not fully differentiate between planned and rescue treatment.	ATG induction; tacrolimus, MMF, and prednisone maintenance; low-dose IVIG after PE	ECU adds PE-based protocols and reduces routine post-transplant PE; there are also age and protocol differences	(15, 19, 20, 36)
West-Thielke 2018	1200 mg 1 day before or on the day of transplantation; 900 mg on days 1, 7, 14, 21, and 28 after surgery; 1200 mg on weeks 5, 7, and 9	4 patients received 10, 3, 3 and 3 pretransplant TPEX respectively, for a total of 19 times	0/4 underwent post-transplant TPEX	ATG induction; IVIG or rituximab not reported; vaccines and short-term antimicrobial prophylaxis	Only reduced post-transplant TPEX, not pretransplant antibody removal	(6, 37)
Heagler 2022	900 mg as a single dose before surgery	Do not schedule pretransplant prophylactic PLEX	Not scheduled for planned PLEX; 1/9 received rescue PLEX after biopsy-confirmed AMR	ATG induction; tacrolimus, MMF, and prednisone; IVIG not reported	A single-dose ECU case series without prophylactic PLEX	(21)
Heo 2024	Case 1: 1200 mg before reperfusion, 900 mg on day 1 after surgery and every week to day 28; Case 2: 1200 mg before reperfusion, 900 mg on days 1, 7, and 14 after surgery; supplementation of 600 mg after PP	Two cases received 6 and 14 pretransplant PPs respectively, combined with rituximab and low-dose IVIG.	Both cases underwent post-transplant PP: Case 1 underwent 8 rescue operations due to suspected AMR, and Case 2 underwent 2 planned rescue operations.	Basiliximab induction; tacrolimus, MMF, and glucocorticoids; Case 1 additionally received high-dose IVIG	Supplementary blockade after intensive desensitization did not reduce overall PP burden	(7)
Mitomo 2025	900 mg as a single dose before reperfusion	Two cases received 6 and 5 albumin-based plasma exchanges respectively.	No post-operative or rescue PLEX reported	Rituximab was used in both cases; high-dose IVIG was used in case 1; basiliximab was used for induction.	Single-session perioperative complement blockade after massive pretransplant replacement	(8)
Yoshikawa 2025	900 mg from 1 day before to the day of transplantation; 900 mg on days 5–7 after surgery	Accessible summary does not report PLEX or other antibody removal	NR	ATG, belatacept, tacrolimus, MMF and prednisone	Two-dose ECU plus belatacept regimen; antibody removal exposes NR	(22)
Okumi 2026	Start 1200 mg initial dose 2 months before transplantation; supplement 600 mg according to CH50 after PE or IVIG; switch to ravulizumab 1 month after transplantation	4 times of pretransplant PE	Postoperative PLEX NR	IVIG 4 g/kg, rituximab, basiliximab; concurrent treatment of suspected aHUS	Individualized complement blockade in the dual risk of AMR and aHUS	(23)
Shimmura 2017	900 mg 5 times weekly, then 3 times every 2 weeks for 8 doses; precise interval between first dose and transplant NR	3 times of DFPP and 5 times of PEX before surgery, a total of 8 times; IVIG and anti-CD20 antibody were also used	No planned post-transplant PLEX reported; AMR treated with steroids and IVIG 6 months later, no rescue PLEX reported	Basiliximab; tacrolimus, MMF, and glucocorticoids	Extended ECU protocol based on intensive pretransplant antibody removal	(24)
NCT01106027	1200 mg 1 hour before surgery; 900 mg on the 1st day after surgery and every week to 4th week; 1200 mg every 14 days from 5th to 9th week if DSA continues to be high and can be extended to 52 weeks	The protocol stipulates 1 PLEX before surgery; NR is actually performed	NR for planned and rescue post-transplant PLEX	Routine transplantation therapy consists of NR; meningococcal, pneumococcal, and Hib vaccines required	The registry results are only 2 cases, and the co-intervention information is incomplete.	(38)
Wan 2026	10 mg/kg pretransplantly; then 5 mg/kg every 3 weeks for 3 months	30/30 in the ECU group received pretransplant DFPP, 1–3 times per case; in the control group, antibody-depleted NR	Staged post-transplant or rescue exposure NR	Control regimens were mainly described for cyclosporine, other co-interventions were incomplete	ECU, DFPP and other joint intervention programs	(39)
Milan 2022/2023	900 mg as a single dose before reperfusion	In the experimental group, each case received PEX once before surgery.	Postoperative PEX 3 times per patient in the 2022 cohort; 3–5 times per patient in the 2023 expansion cohort; historical controls also received pretransplant and post-transplant PEX	IVIG, obinutuzumab, ATG and glucocorticoids	Multi-drug induction based on fixed peri-transplant PEX, ECU and other interventions are administered simultaneously	(40, 41)
NCT06453135	Single dose on planned day of surgery: 900 mg if antibody titer ≥1:64 and/or body weight ≥60 kg, otherwise 600 mg	Program does not plan PLEX	The plan does not plan for post-transplant PLEX; ECU can be added after AMR occurs, rescue PLEX policy NR	Vaccination Requirements; Other Co-Treatments NR	There are no single-dose registration regimens with published results	(34)
ChiCTR2500114806	Administer once 1 day before and 1 week after planned transplantation: 900 mg for those weighing ≥60 kg, otherwise 600 mg	Exclude those who require PLEX or immunoadsorption before surgery	Both planned and rescue post-transplant PLEX were NR	Plan to monitor C5a, C5b-9, CH50, and ECU concentrations; other co-intervention NR	No pretransplant PLEX/immunoadsorption pathway with no published results	(35)

PP/PE/PEX/TPEX all refer to the plasma exchange terms used in the original report; DFPP, double filtered plasma exchange; ATG, antithymocyte globulin; MMF, mycophenolate mofetil; Hib, Haemophilus influenzae type b; NR, not reported in the original report. The number of patients and the number of treatments are listed separately in the table, and planned and rescue PLEX are recorded separately.

### 3.2 Eculizumab dosing and concomitant immunoprophylaxis

ECU dose intensity and treatment duration varied substantially. Marks and Glotz used the same 9-week regimen: 1,200 mg before reperfusion; 900 mg each on post-transplant days 1, 7, 14, 21, and 28; and 1,200 mg each at weeks 5, 7, and 9 (17, 18). The Stegall cohort protocol starts with 1,200 mg before transplantation, and 600 mg per week on post-transplant day 1 and weekly for the first 4 weeks; treatment is extended for patients with persistently high B-cell flow-cytometric crossmatch (15, 19, 20). West-Thielke uses a 9-week multi-dose regimen, while Heagler and Mitomo use a single dose of 900 mg before surgery; Heo, Yoshikawa, Okumi, and Shimmura have different dosing times, intervals, and treatment courses (6–8, 21–24).

Concomitant immunoprophylaxis defined the clinical strategy evaluated in each study. Both groups in the Marks trial received pre-transplant PLEX and/or IVIG as defined by each center, and participants in the intervention group were randomized to receive additional ECU (17). In the Stegall study, recipients in both ECU group and historical controls received pretransplant PLEX, but post-transplant prophylactic PLEX was no longer routinely arranged in the ECU group; this change occurred simultaneously with the treatment era and other clinical management (15). The Glotz study did not routinely schedule pretransplant PLEX or IVIG in the deceased-donor scenario, nor did it schedule planned PLEX in the first 9 weeks, and only allowed rescue therapy after biopsy-proven AMR (18). Most studies on ABO incompatibility still use pretransplant PLEX or double-filtration plasmapheresis (DFPP), combined with IVIG, rituximab, or other immunosuppressive treatments (6–8, 24). The studies therefore evaluated ECU added to different background regimens, rather than ECU monotherapy (**Table 2**).

### 3.3 Comparative evidence for AMR prevention

#### 3.3.1 Randomized comparison

The Marks trial randomized 104 patients, and 102 received transplantation and entered the published analysis, with 51 patients in each group; the grouping and outcomes of the two patients who did not receive transplantation after randomization were not reported. Both groups received pre-transplantation treatment defined by each center. Recipients in the ECU group initiated ECU before reperfusion and continued treatment according to the 9-week protocol. Composite treatment failure at 9 weeks was 5/51 vs. 7/51 (RR 0.71, 95% CI 0.24–2.10; risk difference −39/1,000, 95% CI −171 to 92). Centrally confirmed Banff 2007 grade II/III AMR occurred in 5/51 recipients in each group (RR 1.00, 95% CI 0.31–3.25; risk difference 0/1,000, 95% CI −125 to 125). In the control group, 14/51 received ECU treatment due to AMR determined by the investigator in the first 9 weeks, and a total of 22/51 received ECU over the entire follow-up period. Rescue treatment was guided by clinical assessment and did not necessarily correspond to centrally confirmed AMR. Randomized evidence failed to establish an incremental benefit of ECU for early AMR or composite treatment failure, and the CIs allowed for both clinically important benefit and harm (**Tables 2**, **3**; **Supplementary Table 5**) (17).

**Table 3:** AMR, graft, and kidney-function outcomes.

Study	AMR or rejection definition point in time	AMR and related primary outcomes	Graft and patient outcomes	kidney function	Evidence explanation	References
Marks 2019	Central pathology Banff 2007 Grade II/III AMR; 9 weeks	AMR 5/51 vs 5/51, RR 1.00 (95% CI 0.31–3.25), RD 0 (95% CI −0.125 to 0.125); composite treatment failure 5/51 vs 7/51, RR 0.71 (95% CI 0.24–2.10), RD −0.039 (95% CI −0.171 to 0.092)	Graft loss at 9 weeks 0/51 vs 3/51, RR 0.14 (95% CI 0.01–2.70), RD −0.059 (95% CI −0.159 to 0.021); death 1/51 vs 1/51; KM graft survival at 36 months 91.8% vs 78.5%	No kidney function effects were reported that could be used for consistent comparisons between groups	Neither early AMR nor combined treatment failure showed clear incremental benefit; 22/51 of the control group received ECU crossover during the full follow-up period, and the long-term results reflect a strategy that includes rescue treatment.	(17)
Stegall 2011 and extended follow-up	Deterioration of graft function and clinical AMR consistent with Banff pathology; 3 months	Main cohort 2/26 vs 21/51, RR 0.19 (95% CI 0.05–0.74), RD −0.335 (95% CI −0.482 to −0.129); expansion cohort 2/30 vs 21/48, RD −0.371 (95% CI −0.519 to −0.175)	In the main cohort, 16/16 vs 49/51 had functional grafts at 1 year; in the ECU group, 16/16 only refers to those who met the preset 1-year evaluation of the report and had available data, not the denominator of the entire cohort of 26 cases; in the expansion cohort, the overall graft survival at 5 years was 81.3% vs 82.2%, and at 7 years, 74.1% vs 66.7%	Creatinine in the expansion cohort at 12 months was 1.6±0.9 vs 1.3±0.4 mg/dL; measured GFR was 46.7±16.0 vs 52.6±10.6 mL/min	Early favorable association comes from severely confounded historical comparisons and no durable attributable long-term graft or kidney function benefit was established	(15, 19, 20, 36)
Glotz 2019	Central pathology Banff 2007 Level II/III Clinical AMR; 9 weeks	AMR 3/80, 3.8% (95% CI 0.8%–10.6%); composite treatment failure 7/80, 8.8% (95% CI 3.6%–17.2%)	4/80 grafts were lost and 1/80 died at 9 weeks; KM graft survival was 87.4% at 12 months and 83.4% at 36 months; 6/80 died at 36 months	No standardized comparative kidney-function estimate	There is no concurrent control. The results represent the observed event rate under this plan.	(18)
West-Thielke 2018	Clinical or biopsy proven AMR; follow-up 175–498 days	0/4	4/4 had graft function at their respective final follow-up; only 1 patient reached 1 year	Case reports qualitatively describe functioning as good and do not provide a unified time point summary.	Very small case series, all underwent pretransplant TPEX	(6)
Heagler 2022	Biopsy confirmed AMR; approximately 2 weeks post-transplantly	1/9	8/9 had no rejection and good graft function at last abstract follow-up; fixed time point graft survival rate not reported	NR	AMR still occurs with single-dose regimen without prophylactic PLEX	(21)
Heo 2024	Possible or suspected active AMR as determined by the author; early stage	2/2	12 months 2/2 Graft functional	Creatinine at 12 months was 1.33 and 0.80 mg/dL respectively	Antibody-mediated damage still occurs in ECU superimposed on intensive desensitization	(7)
Mitomo 2025	Clinical or biopsy proven AMR; approximately 1 year	0/2	2/2 grafts are functional	Case 1 had creatinine 1.1 mg/dL at 1 year; case 2 only qualitatively reported good function	Both cases received 5–6 pretransplant PLEX, rituximab, and other co-interventions	(8)
Yoshikawa 2025	Accessible summary undefined AMR	No AMR events or diagnoses reported	Graft loss and death both NR	eGFR≥40 mL/min/1.73 m² for 3/3 of 1 month	Only short-term kidney function and infection outcomes are reported, AMR outcomes are missing	(22)
Okumi 2026	3-month and 1-year protocol biopsies	No AMR was found in 3-month and 1-year biopsies (1 case)	The graft is functional and the kidney function is stable at 1 year	0.87 mg/dL on the 14th day after surgery, 1.18 mg/dL at 1 month	ECU is used for both AMR prevention and suspected aHUS treatment. The prevention effect alone cannot be judged.	(23)
Shimmura 2017	Biopsy-confirmed AMR; post-transplant day 14 and 6 months later	0/1 on the 14th day; 1/1 after 6 months	Graft loss and death both NR	Creatinine 1.5 mg/dL on day 14	Early negative observations do not exclude late-onset AMR	(24)
NCT01106027	Banff 2005 Acute humoral rejection; 1 year	Main outcome module reports 0/1, serious adverse event module reports 1/2	1/2 early graft loss; 0/2 died; 1/2 completed study	NR	Primary outcome based on 1 completer, safety module based on 2 recipients	(38)
Wan 2026	Undefined rejection; 3 months	1/30 vs 3/30; no AMR identifiable by transplant pathology criteria was reported	The study-reported transplantation success rate was 27/30 versus 21/30; the component events were not defined	Creatinine 2.4±0.5 vs 1.0±0.3 mg/dL, which is opposite to the favorable narrative direction in the article	There are internal inconsistencies in groupings, denominators, outcome definitions, and direction of results, and the interpretability of comparative results is low.	(39)
Milan 2022/2023	AMR definition is incomplete; median follow-up 13 or 16 months	2022 Abstract 2/7; 2023 Extended Abstract 0/10 vs 7/20, the two versions conflict	2022 cohort 7/7 had functional grafts; 2023 abstract AMR-related graft losses 0/10 vs 4/20, 0/10 deaths in experimental group	The last median creatinine of the experimental group: 1.6 (1.1–2.5) in 2022 and 1.3 (0.8–2.5) mg/dL in 2023	Version conflicts and multi-drug regimens are not interchangeable with historical comparisons	(40, 41)

The main cohort and extended follow-up of the same study in the table are indicated separately by source, and overlapping recipients are not counted repeatedly. KM, Kaplan–Meier; GFR, glomerular filtration rate; NR, not reported in the original report. Rejection in Wan's study is not equivalent to AMR; 0/1 and 1/2 of NCT01106027 are from different outcome modules.

#### 3.3.2 Historical and other nonrandomized comparisons

In Stegall’s historical comparison, 3-month clinical AMR occurred in 2/26 recipients in the ECU group and 21/51 historical controls (RR 0.19, 95% CI 0.05–0.74; risk difference −335/1,000, 95% CI −482 to −129). Routine post-transplant prophylactic PLEX was discontinued in the ECU group, whereas historical controls received 4 to 14 post-transplant PLEX, and treatment eras and clinical management differed, so this association cannot isolate the independent effect of ECU (15).

Wan reported undefined rejection events in 1/30 and 3/30, but the grouping method, AMR definition, and co-intervention were insufficient to support an effect estimate. The 30 cases/group in the main text and the 70 cases/group in some tables also conflicted with each other (39). The Milan project used a multidrug regimen consisting of ECU, obinutuzumab, IVIG, ATG, and fixed peri-transplant PLEX, and compared it with the historical rituximab regimen; two overlapping abstracts reported AMR 2/7 and 0/10, respectively, and conflicting data and versions hindered comparative interpretation (40, 41). These studies retain the original results and are not used to establish the comparative effect of ECU (**Table 3**).

### 3.4 AMR observations in uncontrolled studies

The Glotz single-arm study enrolled 80 deceased-donor kidney transplant recipients with preexisting DSA and reported 9-week Banff grade II/III AMR 3/80, or 3.8% (95% CI 0.8%–10.6%), and composite treatment failure 7/80, or 8.8% (95% CI 3.6%–17.2%), in a regimen that did not routinely schedule pretransplant PLEX or IVIG. The 40% expected event rate in the scenario is not a contemporaneous control and cannot be converted into a comparative effect (18).

Studies of ABO-incompatible living donor kidney transplantation have had small sample sizes. No early confirmed AMR was observed in four cases at West-Thielke and two cases at Mitomo, but all recipients underwent pretransplant antibody removal (6, 8). Heagler used a single pretransplant ECU and did not arrange prophylactic PLEX. One of the nine cases developed biopsy-proven AMR and received rescue PLEX (21). Both recipients reported by Heo developed possible or suspected active AMR after intensive pretransplant desensitization and ECU, and a single case of Shimmura developed biopsy-confirmed AMR at 6 months (7, 24).

Other small studies provide even more incomplete data on AMR. NCT01106027 reports AMR in 0/1 and 1/2 recipients in different results modules, with inconsistent denominators (38). The Yoshikawa report did not define AMR or report its frequency among the three recipients (22). In the case reported by Okumi, ECU was given for both AMR prevention and suspected aHUS, limiting its relevance to AMR prophylaxis alone (23). These uncontrolled studies describe AMR events under different regimens. Differences in clinical setting, diagnostic criteria, biopsy strategy, and follow-up precluded pooling of AMR rates, and the absence of controls precluded comparison with standard treatment (**Table 3**).

### 3.5 Graft, patient, and kidney-function outcomes

In the Marks randomized trial, graft loss by week 9 occurred in 0/51 recipients in the ECU group and 3/51 in the standard-desensitization group (RR 0.14, 95% CI 0.01–2.70; risk difference −59/1,000, 95% CI −159 to 21). Three cases of graft loss in the control group were attributed to technical complications, acute AMR, and graft vascular dysfunction; by 12 months, two more cases of graft loss occurred in the ECU group, and one additional loss occurred in the control group (17). By week 9, one recipient had died in each group (1/51 versus 1/51; RR 1.00, 95% CI 0.06–15.56; risk difference 0/1,000, 95% CI −85 to 85). Estimated patient survival at 36 months was 98.0% in each group. At 36 months, estimated graft survival was 91.8% in the ECU group and 78.5% in the standard-desensitization group, with no statistically significant between-group difference (17). The point estimate for short-term graft loss favored ECU, but events were few and CIs allowed for both significant benefit and mild harm, and patient survival showed no difference between groups.

Interpretation of the long-term Marks trial results is complicated by rescue ECU use in the control group. Of 51 patients in the standard desensitization treatment group, 22 received at least one dose of rescue ECU during follow-up, and 14 of them received treatment for AMR in the first 9 weeks (17). The original article reported estimated glomerular filtration rate and serum creatinine at baseline, 9 weeks, and 12 months, but did not provide long-term kidney function estimates excluding rescue crossover effects. The 36-month graft survival rate thus reflects the follow-up results of a treatment strategy that included rescue ECU rather than the effects of prophylactic ECU alone.

The Stegall historical comparison and its extended follow-up provide the longest comparative graft and kidney function data. In early reports, 16/16 ECU recipients who met the prespecified 1-year evaluation and for whom data were available retained functional grafts, compared with 49/51 historical controls; 16/16 was not the complete denominator of the original 26 ECU cohort (15). During extended follow-up, the 12-month serum creatinine of the two groups was 1.6 ± 0.9 and 1.3 ± 0.4 mg/dL, respectively, and the measured GFR was 46.7 ± 16.0 and 52.6 ± 10.6 mL/min. There was no significant difference between the groups; the 2-year death-adjusted graft survival rate was 89.7% and 95.8% (19). In a longer-term report, the overall graft survival rates of the ECU group and the positive crossmatch historical control group were 81.3% and 82.2% at 5 years, and 74.1% and 66.7% at 7 years. There was no significant difference in the overall and death-adjusted graft survival curves; the median serum creatinine of those with functional grafts at 5 years was 1.5 and 1.3 mg/dL, respectively (20). These serial follow-ups did not show stable translation of early AMR differences into long-term graft survival or kidney function advantages.

In the Glotz single-arm study, graft loss increased from 4/80 at 9 weeks to 10/80 at 12 months. Kaplan–Meier patient survival rates were 97.5% and 91.5% at 12 and 36 months, respectively, and graft survival rates were 87.4% and 83.4% (18). Thirteen of the 76 evaluable recipients experienced delayed graft function; according to the different functional outcome denominators provided by the study, 4/48 required dialysis from 7 days to 12 months after transplantation. Serum creatinine follow-up was recorded but no concurrent controls were available. This cohort describes patient, graft, and kidney function outcomes 3 years after implementation of a deceased-donor ECU regimen and does not provide comparative effects relative to a strategy without ECU.

Small ABO incompatibility studies primarily provide short-term functional observations at the individual level. All four West-Thielke cases had functioning grafts at the last follow-up of 175 to 498 days, but only one case reached 1-year follow-up (6). Serum creatinine in two cases of Heo at 12 months was 1.33 and 0.80 mg/dL, respectively, and both cases retained functional grafts; both cases had previously received additional PLEX or other treatments due to suspected antibody-mediated injury (7). Both cases of Mitomo reported good graft function approximately 1 year later, and one of them had a serum creatinine of 1.1 mg/dL (8). The eGFR of Yoshikawa’s three cases was not less than 40 mL/min/1.73 m² at 1 month, and the serum creatinine of Okumi’s single case on 14 days and 1 month after surgery was 0.87 and 1.18 mg/dL respectively (22, 23). Graft function was preserved over short follow-up in these reports, showing that transplantation with these combination regimens was feasible. The very small samples, variable follow-up, and absence of concurrent controls limit conclusions about comparative benefit.

The remaining data were limited by inconsistencies in outcome definitions and reporting. Wan et al. reported transplantation success in 27/30 and 21/30 recipients, but did not define the component events of this outcome. The study also reported serum creatinine of 2.4 ± 0.5 and 1.0 ± 0.3 mg/dL, which is inconsistent with the author’s description of better recovery in the ECU group (39). In the Milan abstract, 7/7 functional grafts were reported in 2022, and 0/10 and 4/20 AMR-related graft losses were reported in the 2023 expanded version, but the numbers of AMR events in the two versions conflicted with each other, and multi-drug regimens and historical controls were used (40, 41). Based on comparative and non-comparative evidence, existing studies have not established the incremental benefit of ECU on patient survival, long-term graft survival, or kidney function; the more consistent observation is that most recipients in small series still retain functional grafts during short-term follow-up (**Table 3**).

### 3.6 DSA, complement pharmacodynamics, and kidney-allograft pathology

HLA-related studies did not provide complete longitudinal DSA trajectories for group-level comparison. Marks and Glotz reported baseline DSA and some follow-up measurements but did not provide continuous changes that could be used for consistent comparisons between groups (17, 18). Lefaucheur re-stratified the Marks and Glotz data by C1q combined with DSA; 3-month biopsy confirmed that AMR in C1q-positive recipients was 18/32 in the standard desensitization treatment group and 7/37 in the ECU group. There were also inter-group differences in glomerulitis and peritubular capillaritis scores (16). This analysis selectively combined the subgroups of a randomized trial and a single-arm study without retaining the original randomized comparison structure. Therefore, it suggested the relationship between complement fixation ability and early injury phenotype, rather than the clearance effect of ECU on DSA. The B54 DSA MFI of the second patient in the Heo study dropped from 2,607 at baseline to 2,327 before transplantation and 346 at 12 months, but he also received rituximab, 14 pretransplant PLEX sessions, 2 post-transplant PLEX sessions, and other immunosuppressive treatments during the same period (7). HLA-related data did not show a consistent decrease in DSA; the decrease in the Heo single case occurred in the context of the combined use of multiple antibody removal and immunotherapy.

ABO incompatibility studies provide a more complete blood group antibody time series. West-Thielke reported four cases of IgG-type ABO antibodies measured by antiglobulin test, recording titers before and after pretransplant PLEX and at 1 day, 1 week, and 1 to 12 months after surgery (6). After patient 1 received 10 pretransplant PLEX sessions, the titer dropped from 1:64 to 1:32; after transplantation and starting ECU, the titer rebounded to 1:1,024 1 week after surgery, rose to 1:2,048 after 1 month, and remained 1:512 at 6 and 12 months. This patient did not develop clinical AMR, but protocol biopsies continued to show diffuse C4d positivity. The remaining three patients received transplantation with titers ranging from 1:2 to 1:32 after three pretransplant PLEX sessions, with overall low post-transplant titers and variable follow-up completeness. This group of cases illustrates that IgG-type ABO antibodies can persist or rebound during ECU treatment, but antibody titers, C4d deposition, and clinical AMR are not necessarily synchronized.

The Heo study reported IgM and IgG anti-B antibodies in two patients from before PLEX to 12 months after surgery (7). Case 1 dropped from 1:512/1:512 to 1:16/1:128–1 day before transplantation after six pretransplant PLEX sessions; it was 1:64/1:64–7 days after surgery, and dropped to approximately 1:8 to 1:16 at 1 to 3 months. Case 2 dropped from 1:256/>1:1,024 to 1:4/1:64 after 14 pretransplant PLEX sessions; it was 1:2/1:32 1 day after surgery, and then fluctuated to 1:16/1:64 at 12 months. Two cases received eight and two additional post-transplant PLEX sessions, respectively, and were combined with IVIG, rituximab, glucocorticoids, and other immunosuppressive treatments. The admission anti-A/B antibody titers of the two cases studied by Mitomo were 1:32,768 and 1:2,048 respectively. After five to six times of pretransplant albumin-based PLEX, rituximab, and partial IVIG treatment, they were still 1:256 at the time of transplantation. After a single ECU before reperfusion, the authors reported that the post-transplant anti-blood group antibodies remained below 1:2 (8). This study mainly used indirect antiglobulin test to detect IgG-type antibodies and did not provide the same complete IgM sequence. The low titers in both studies occurred after multiple antibody depletions and combined immunotherapy, thus documenting antibody changes after protocol implementation but not isolating the independent effect of ECU.

The remaining ABO-incompatible reports provide incomplete longitudinal antibody data. The baseline anti-A IgG and IgM of the Shimmura case were 1:4,096 and 1:512, respectively, and decreased after DFPP, PLEX, IVIG, and anti-CD20 treatment, but the accessible report did not provide a complete serial sequence before and after ECU administration; the recipient had no AMR in early biopsy, and biopsy confirmed that AMR occurred 6 months later (24). Wan’s study reported changes in IgM, IgG, and anti-A/B antibodies, but the relevant table marked 70 cases/group, which was inconsistent with the 30 cases/group in the main text; thus, its numerical values were not used to explain the antibody changes (39). Antibody changes in these studies were observed in the setting of combined desensitization and post-transplant immune adaptation. The independent effect of ECU on IgG or IgM remains unclear.

Pathological results further indicate that terminal complement blockade, upstream complement activation, and chronic microcirculatory injury need to be evaluated separately. In the Stegall cohort and its extended follow-up, persistent DSA, microvascular inflammation, and transplant glomerulopathy were still observed despite less early clinical AMR; 12-month transplant glomerulopathy was 1/15 and 15/42 in the early report, and glomerulitis, peritubular capillaritis, and C4d positivity were still seen in 5-year biopsy in the expanded cohort (15, 19, 20). This group of consecutive follow-ups did not show stable translation of early terminal complement inhibition into long-term histological improvement. Two cases of Mitomo showed strong C4d deposition during reperfusion and follow-up biopsy, but neither biopsy showed corresponding microvascular inflammation or clinical AMR (8). Suppression of CH50 during ECU treatment was observed in the Heo and Okumi cases, but this pharmacodynamic change was not accompanied by the simultaneous elimination of antibodies or all histological abnormalities (7, 23). Based on existing research, HLA-DSA and ABO antibodies can decrease, persist, or rebound during ECU treatment, and terminal complement inhibition has not stably eliminated C4d deposition, microvascular inflammation, or transplant glomerulopathy. Antibody burden, complement pharmacodynamics, histology, and clinical AMR reflect outcomes at different levels and should be reported separately (**Table 4**).

**Table 4:** DSA, kidney-allograft pathology, and safety outcomes.

Study	DSA, ABO antibody or complement results	Main pathological results	Serious adverse events and infections	Evidence explanation	References
Marks 2019	Reports baseline DSA but does not provide sequential post-transplant DSA changes comparable by randomization group	9 Weeks Central pathology Banff 2007 Grade II/III AMR 5/51 vs 5/51	Serious adverse events 43/51 vs 48/51; serious infections 32/51 vs 25/51; meningococcal infection 0/51 vs 0/51	Only randomized safety comparison; sample size insufficient to evaluate rare meningococcal infections	(17)
Glotz 2019	Continuous DSA monitoring was performed, but no longitudinal pooled effects were available	Central pathology AMR at 9 weeks was 3/80; local pathology was judged to be 7/80	Serious adverse events at 36 months 70/80; study-defined clinically important infections 65/80	Single-arm long-term event spectrum, without control group Estimating incremental infection risk in ECU	(18)
Stegall 2011 and extended follow-up	High DSA recipients had 13/13 vs 20/22 positive biopsies for C4d within 3 months; longitudinal DSA results across cohort heterogeneous across reports	12-month transplant glomerulopathy 1/15 vs 15/42; expansion cohort 26.7% vs 39.5% at 12 months, 45.4% vs 63.6% at 24 months; 5-year glomerulitis 12/19 vs 16/28, peritubular capillaritis 9/19 vs 12/28, C4d positivity 6/19 vs 2/28	The main report only lists 1/26 wound infections in the ECU group; the number of historical control infections is NR; the definition of safety in long-term reports is not uniform	Early clinical AMR signal does not eliminate chronic microcirculatory injury; pathological denominators are all evaluable biopsy subgroups	(15, 19, 20, 36)
Lefaucheur post hoc analysis	Stratified by C1q combined with DSA; C1q positive group was SOC 32 cases and ECU 37 cases	For SOC versus ECU, the 3-month ABMR of all DSA-positive recipients was 21/64 vs 9/52; the C1q-positive recipients were 18/32 vs 7/37, and the C1q-negative recipients were 3/32 vs 2/15; the median glomerulitis score on day 14 in the C1q-positive group was 2 vs 1, and peritubular capillaritis was 2 vs 0.	No independent safety population	Post hoc analysis with selective merging of Marks and Glotz subgroups without retaining the preset randomized comparison structure	(16)
West-Thielke/Campara 2018	ABO-IgG titer decreased from 1:16–1:2048 before surgery to 1:2–1:32 before transplantation; serial DSA data NR	Reported clinical or biopsy AMR within follow-up 0/4; biopsy surveillance incomplete	Any infection 2/4; drug-resistant CMVemia 1/4; meningococcal infection 0/4	The efficacy and infection data are from the same 4-case cohort.	(6, 37)
Heagler 2022	Individual changes in ABO titers reported; complete serial DSA results NR	Biopsy confirmed AMR 1/9, complete Banff grade NR	Any infection 6/9	An abstract-level case series without prophylactic PLEX, infection and AMR affected by co-immunosuppression	(21)
Heo 2024	The B54 DSA MFI of Case 2 dropped from 2607 to 2327 before transplantation and to 346 at 12 months; both cases had changes in complement or immune-related molecular phenotypes.	Both cases were what the authors judged to be possible or suspected active AMR.	Major clinical infection 0/2; BK DNAemia 1/2	DSA or molecular changes do not translate into clear AMR prevention	(7)
Mitomo 2025	Background of extremely high ABO antibody titers; no summarizable post-transplant DSA changes reported	Strong C4d deposition was seen in both cases without corresponding microvascular inflammation or clinical AMR.	Serious adverse events and infections: NR	C4d positivity itself does not equate to clinical AMR	(8)
Yoshikawa 2025	Two-thirds of type I and type II DSA decreased, and one-third had all DSA disappeared at 2 months.	Accessible abstracts did not report analyzable biopsy results or AMR events	Bacterial infection, CMVemia and BKemia within 6 months were all 0/3	Immunological and short-term safety observations in very small samples	(22)
Okumi 2026	DSA qualitatively decreased after desensitization; CH50 was lower than the detection limit on the day of transplantation.	No AMR in 3-month and 1-year protocol biopsies	Infection outcome within 1 year NR	ECU, PLEX, IVIG, and rituximab used together to treat suspected aHUS	(23)
Shimmura 2017	Baseline anti-A IgG/IgM was 1:4096/1:512, which decreased after intensive desensitization; accurate post-transplant sequence was incomplete	No AMR on day 14, biopsy confirmed AMR 6 months later	Infection outcome NR	A single case shows that early negative pathology and late-onset AMR can coexist	(24)
NCT01106027	The plan is to extend ECU according to DSA decision, but the result is not announced DSA trajectory	Main Module 0/1 Acute Humoral Rejection; Serious Adverse Event Module 1/2 Antibody-Mediated Rejection	Serious adverse events 1/2; non-serious adverse events 2/2; pneumonia 1/2; death 0/2	The result modules use different denominators and must be presented side by side.	(38)
Wan 2026	The IgM, IgG and anti-A/B drop values are from the conflict table marked 70 cases/group in the header, without quantitative explanation.	No biopsy, Banff criteria, or adjudicated AMR reported	Any complication 2/30 vs 8/30; infection 1/30 vs 4/30; the counts and percentages of some items are inconsistent	Internal inconsistencies in denominators and counts make safety comparisons less reliable	(39)
Milan 2022/2023	2022 Abstract Report de novo DSA 0/7; inconsistent anti-HLA antibody changes	The 6-month protocol biopsy in 2022 and the biopsy within 6 months after obinutuzumab in 2023 were both reported to have no AMR features, and the biopsy denominator was NR	2022: CMV 4/7, EBV 1/7, BK 0/7; 2023: life-threatening infection 0/10 vs 2/20, severe leukopenia 2/10 vs 1/20	Early 7 cases may be included in the expanded 10 cases; multidrug regimens and reporting conflicts limit attribution of ECU effects	(40, 41)
Brealey 2018	No interpretable consecutive DSA or complement outcomes reported	Serial biopsies from a single case describe focal peritubular capillary basement membrane multilayering, lipid droplets, and apoptotic changes	Safety outcome NR	Public sources fail to confirm its relationship to other included cohorts	(33)

The denominator of pathological results is the evaluable biopsy samples in the original study, not the entire treatment cohort. C1q, complement component 1q; CMV, cytomegalovirus; EBV, Epstein–Barr virus; CH50, total complement hemolytic activity; NR, not reported in the original report.

### 3.7 Safety

The Marks randomized trial provides the only directly comparable safety data. Serious adverse events that occurred during treatment in the ECU group and the standard desensitization treatment group were 43/51 and 48/51 (RR 0.90, 95% CI 0.78–1.03; risk difference −98/1,000, 95% CI −227 to 27), of which serious infections were 32/51 and 25/51 (RR 1.28, 95% CI 0.90–1.82; risk difference 137/1,000, 95% CI −54 to 315) (17). Urinary tract infection, pyelonephritis, urosepsis, and pneumonia were all seen in both groups, and individual infection types showed no consistent pattern. One patient in the ECU group died of bacterial sepsis on the 38th post-transplant day. The researchers believed that the infection may be related to ECU and was also attributed to intestinal perforation after renal biopsy and post-transplantation immunosuppression load. One patient in the control group died of cardiac arrest on the 5th post-transplant day. No meningococcal infections occurred in either group, but the double-zero results of 51 cases each were insufficient to estimate rare risks. The total number of serious adverse events did not show an increase in the ECU group, but the point estimate for serious infections favored an increased risk, with CIs that still allowed for both no difference and a clinically meaningful increase.

The Glotz single-arm study showed the absolute event burden over longer follow-up. All 80 cases had at least one adverse event during treatment; 70/80 had serious adverse events within 36 months, of which 5/80 were judged to be drug-related serious adverse events, and 65/80 had confirmed clinically important infections as defined by the study (18). Anemia, diarrhea, and transplant rejection including various immunological biopsy manifestations are the most common adverse events. This event spectrum arose in the clinical context of ECU, maintenance immunosuppression, high immunologic risk status, and post-transplant exposure.

ABO incompatibility and other small studies have reported different types of infections. Two of the four cases in the West-Thielke/Campara cohort developed infections, and one of them developed *Clostridium difficile* infection and ganciclovir-resistant CMVemia; this patient was a high-risk combination of CMV IgG-positive donor and negative recipient, and CMVemia occurred approximately 3 months after stopping ECU (6, 37). The Heagler abstract reported that infection occurred in six of nine cases, but did not provide enough data to uniformly determine the severity (21). No major clinical infection occurred in the two cases of Heo, and one of them developed BK DNAemia at 3 months and had to adjust immunosuppressive therapy (7). None of the three cases in Yoshikawa reported bacterial infection, CMVemia, or BKemia within 6 months (22). These positive and zero events are study-specific observations in extremely small samples and do not form stable incidence estimates.

The remaining sources are further limited by denominator, definition, or cohort overlap. NCT01106027 reported one serious adverse event and one pneumonia among two treated patients, but the denominators used by the different outcome modules were inconsistent (38). Wan reported infections on 1/30 and 4/30. There were internal contradictions in some of the complication counts and percentages and the grouping information in the full text. This review retains the original event description (39). Milan reported CMV 4/7, EBV 1/7, and BK 0/7 in the early 7 cases; the extended abstract reported life-threatening infections 0/10 and historical controls 2/20, but the early 7 cases may be included in the extended 10 cases, and ECU was used simultaneously with multi-drug induction such as obinutuzumab (40, 41). These results are presented separately as safety events for each study and are not combined with the Marks randomized trial.

Based on the existing evidence, the randomized trial did not show an increase in the total number of serious adverse events in the ECU group, but the estimate of serious infections was imprecise and still allowed for an increase in risk. Events such as CMV, BK, EBV, bacterial infection, and pneumonia constituted the spectrum of safety events in single-arm studies and case series; the incremental infection risk associated with ECU was still defined by the wide interval provided by the Marks trial (**Table 4**; **Supplementary Table 6**).

### 3.8 Risk of bias

The composite treatment failure and central pathology AMR of the Marks trial were both rated as RoB 2 “with some concerns”, mainly based on the fact that only 102 of 104 cases were included in the published analysis after randomization, the grouping of two non-transplant patients was not reported, and the safety comparison of the entire study period was affected by the rescue crossover use of ECU in the control group (17). Stegall’s historical comparison rated ROBINS-I as having a critical risk of bias due to differences in treatment era, post-transplant PLEX and clinical management, lack of confounding adjustment, and differences in follow-up (15). The Wan study cannot be used to estimate the treatment effect of ECU due to the lack of grouping methods, confounding control and rejection definitions, and the 30/70 denominator conflict (39). Milan’s two overlapping abstracts have both historical confounding and AMR conflicts of 2/7 and 0/10, and are not used for treatment effect estimation (40, 41).

The enrollment, ECU exposure, and follow-up reports of the Glotz single-arm study are relatively clear, but there is a lack of concurrent controls, and the indications for rescue treatment are also insufficiently reported, so it can only describe the event rate after the implementation of the protocol (18). The Chicago, Heo, and Mitomo case series all have problems such as strong potential for case-selection bias, unclear consecutive or complete inclusion, and intensive co-interventions (6–8, 21, 37). Yoshikawa and Shimmura only have conference abstracts, with less evaluable methods and outcome information; Okumi is a single case and the indications for AMR prevention and aHUS cannot be separated (22–24). Apart from the Marks randomized trial, the available studies describe treatment regimens, clinical events, and longer-term outcomes, but do not provide comparative effect estimates at low risk of bias (**Supplementary Table 4**).

## 4 Discussion

This review distinguishes between the clinical feasibility and comparative efficacy of ECU. ECU has been added to the peri-transplant regimen for HLA-incompatible living donor transplantation, pre-existing DSA deceased-donor transplantation and ABO-incompatible transplantation, but only the Marks trial directly evaluated its incremental effect on established background therapy (17). This trial did not establish the benefit of ECU in reducing early AMR or composite treatment failure. Stegall historical comparison and Glotz single-arm studies provide early event and long-term follow-up data under different protocols, but cannot support the same causal interpretation as randomized trials (15, 18). Case reports and single-arm studies document clinical use and outcomes in selected recipients, while the randomized comparison provides the strongest evidence on efficacy.

The significance of the Marks trial goes beyond the fact that the primary endpoint did not show a clear difference between groups. It compares the planned addition of 9 weeks of ECU to each center’s desensitization program, rather than the comparison of ECU alone and no treatment (17). The differences between central pathology determination, local clinical AMR diagnosis, and rescue treatment illustrate that pathological endpoints and bedside treatment decisions reflect different issues. Subsequent rescue ECU use in the control group also complicates interpretation of long-term differences between the randomized groups. The trial did not establish the incremental efficacy of ECU, nor did it prove that the two strategies were equivalent; the current sample size and crossover treatment make clinical effect estimates still imprecise.

Observational studies explain why early favorable signals did not translate into consistent conclusions. The Stegall study changed post-transplant PLEX and other clinical management while introducing ECU, and what was observed was the combined result of the entire treatment strategy and changes over time (15). The Glotz study demonstrated a 9-week ECU pathway in a deceased-donor scenario where predesensitization was not possible, but the preset event rate was not a concurrent control (18). Wan and Milan further documented the practice of ECU combined with DFPP, obinutuzumab, and other immune interventions, and the consistency of grouping, outcome definition, and reporting was insufficient to support reliable comparisons (39–41). These studies describe outcomes and rescue treatment under combination regimens, but do not establish the benefit attributable to ECU.

ECU dose, timing, and treatment duration also varied across studies. Marks and Glotz use a fixed 9-week multi-dose regimen, and the Stegall regimen can extend treatment based on B-cell flow-cytometric crossmatch (15, 17–20). West-Thielke uses multiple dose courses, and Heagler and Mitomo give a single dose of 900 mg before transplantation or reperfusion (6, 8, 21). Heo, Yoshikawa, Okumi, and Shimmura also used different administration times and intervals (7, 22–24). Existing studies have not directly compared single versus multiple administrations, nor have they established a dose–response relationship between CH50 inhibition duration, ECU exposure, and clinical outcomes. Therefore, different ECU dosing regimens should not be regarded as the same intervention with uniform effects.

The pharmacologic target of ECU provides a rational explanation for the dissociation of short-term clinical signals from long-term pathology. Blocking C5 reduces terminal complement damage mediated by C5a and C5b-9 but does not clear DSA and does not block upstream C3 activation and complement-independent antibody effects (5, 13, 16). In the long-term follow-up of the Stegall cohort, persistent DSA, microvascular inflammation, and transplant glomerulopathy were still observed, and no consistent advantage emerged in graft survival and kidney function (19, 20). Antibody rebound, persistent C4d positivity, and discordance with clinical AMR in ABO incompatibility cases suggest that blood group antibodies, complement deposition, and tissue damage are also affected by immune adaptation and other factors (6, 8). These findings are consistent with the explanation that ECU mainly affects the early terminal complement effect stage, but does not reduce the antibody load or block all antibody-mediated damage.

High-immunologic-risk kidney transplantation does not represent a single clinical setting. Living-donor HLA-incompatible transplantation permits pretransplant testing and planned desensitization, whereas deceased-donor transplantation allows less preparation time and ABO-incompatible transplantation has distinct antibody kinetics and histologic interpretation (6–8, 17, 18). Therefore, event rates in one scenario cannot be directly extrapolated to another scenario. Lefaucheur’s *post-hoc* analysis suggested that C1q combined with DSA may identify phenotypes with more active terminal complement damage, but this analysis did not retain the original random comparison structure (16). Existing studies have not established immunological thresholds, pathological phenotypes or clinical selection criteria that can predict the benefit of ECU.

Serious infection is a safety outcome that needs to be explained in the existing randomized evidence. There was no increase in the overall number of serious adverse events in the randomized trials, but the estimate for serious infection remained compatible with a clinically important increase (17). CMV, BK, EBV, and bacterial infections in single-arm studies and case data occurred in the shared context of intensive induction, maintenance immunosuppression, and antibody removal and cannot be attributed to a single agent (6, 7, 18, 21, 37). Meningococcal infection was not reported in the included studies, but these samples are insufficient to evaluate rare events; vaccination and necessary antimicrobial prophylaxis should still follow product information and public health guidance (14, 42, 43). Clinical safety evaluation should cover the entire immunoprophylaxis regimen, not just record ECU infusion-related events.

KDIGO and ESOT prioritize the acquisition of compatible donor kidneys through allocation, acceptable mismatch, or kidney pair exchange, and use desensitization based on antibody removal when antibody barriers must be crossed (1, 2). The ESOT 2024 ENGAGE consensus did not reach the consensus threshold for using complement inhibitors for the prevention of high-risk AMR, and the 2025 Chinese Guidelines included ECU in an individualized combination pretransplant desensitization pathway (29, 30). Product information in the United States and Europe has not yet listed AMR prevention in kidney transplantation as an indication (14, 42). Existing evidence does not support the use of ECU as routine preventive treatment for all high-immunologic-risk kidney transplant recipients; if used in specific recipients, it should be within the framework of a clear combination regimen, infection prevention, and prospective outcome monitoring.

The strengths of this review include merging multiple reports by study project, identifying cohort overlap, and treating central pathology AMR, local clinical judgment, composite treatment failure, and rescue treatment separately (**Supplementary Table 3**). The main limitation is that the comparative evidence consists of only one small randomized trial and one severely confounded historical comparison. Other data are mostly single-arm studies, case reports, conference abstracts, or registries with incomplete results. The main comparative study and the largest single-arm study received Alexion funding or research support, and there was less independent replication evidence (15, 17, 18).

Subsequent direct trials should focus on one clinical scenario and separately study living HLA-incompatible, deceased-donor pre-existing DSA, or ABO-incompatible recipients, rather than combining these populations in one efficacy estimate. The trial should have fixed background desensitization, induction, and maintenance of immunosuppression, and pre-specify triggering conditions for rescue ECU and PLEX. The primary analysis should retain the randomly assigned treatment strategy and report rescue therapy as a prespecified outcome; central interpretation of AMR, local diagnosis, and composite treatment failure should also be presented separately (44). Follow-up should simultaneously measure DSA and its complement fixation characteristics, C4d, microvascular inflammation, transplant glomerulopathy, kidney function, graft survival, and infection (44, 45). Such a design would more directly determine whether ECU improves patient-important outcomes and whether its clinical benefits are sufficient to offset the risk of infection and treatment burden.

## Data Availability

The raw data supporting the conclusions of this article will be made available by the authors, without undue reservation.
