# Supplementary material for: Peri-transplant eculizumab for preventing antibody-mediated rejection in kidney transplant recipients at high immunologic risk: a systematic review with design-stratified evidence synthesis
Source: Front Immunol. 2026 Oct 20;17:1982870. doi: 10.3389/fimmu.2026.1982870 (PMC13727399; doi:10.3389/fimmu.2026.1982870)
Supplement: Supplementary file 1 [file DataSheet1.docx]

**Supplementary Table S1. Complete search strategies for databases and other sources**

| **Source/Platform** | **Coverage** | **Search date** | **Complete search query or execution method** | **Number of hits** |
| --- | --- | --- | --- | --- |
| PubMed/MEDLINE (main search) | Database coverage through 2026-06-10 | 2026-06-10 | (eculizumab[tiab] OR Soliris[tiab] OR eculizumab[nm]) AND ("Kidney Transplantation"[Mesh] OR "kidney transplant"[tiab] OR "kidney transplantation"[tiab] OR "kidney transplants"[tiab] OR "kidney transplant"[tiab] OR "kidney transplantation"[tiab] OR "kidney transplants"[tiab] OR "kidney allograft"[tiab] OR "kidney allografts"[tiab] OR "renal allograft"[tiab] OR "renal allografts"[tiab] OR "kidney graft"[tiab] OR "kidney grafts"[tiab] OR "renal graft"[tiab] OR "renal grafts"[tiab]) | 458 |
| PubMed/MEDLINE (supplementary search) | Database coverage through 2026-06-10 | 2026-06-10 | (eculizumab[tiab] OR Soliris[tiab] OR eculizumab[nm]) AND (transplant*[tiab] AND ("antibody-mediated rejection"[tiab] OR "antibody mediated rejection"[tiab] OR "antibody-mediated rejection"[Mesh] OR AMR[tiab] OR ABMR[tiab] OR sensitiz*[tiab] OR desensitiz*[tiab] OR "donor-specific antibod*"[tiab] OR "donor specific antibod*"[tiab] OR crossmatch*[tiab] OR "cross match"[tiab] OR "cross-match"[tiab])) | 177 |
| Ovid Embase | 1974 to 2026-06-05 | 2026-06-10 | 1. exp Eculizumab/; 2. (eculizumab or soliris).ti,ab,kw.; 3. 1 or 2; 4. exp Kidney Transplantation/; 5. ((kidney or renal) adj3 (transplant* or allograft* or graft*)).ti,ab,kw.; 6. 4 or 5; 7. 3 and 6 | 1,751 |
| Web of Science Core Collection | All years；All editions | 2026-06-10 | TS=(eculizumab OR Soliris) AND TS=((kidney OR renal) NEAR/3 (transplant* OR allograft* OR graft*)) | 685 |
| Ovid CENTRAL | Until June 2026 | 2026-06-10 | 1. exp Kidney Transplantation/; 2. ((kidney or renal) adj3 (transplant* or allograft* or graft*)).ti,ab,kw.; 3. 1 or 2; 4. (eculizumab or soliris).ti,ab,kw.; 5. 3 and 4 | 35 |
| ClinicalTrials.gov (Conditions & Interventions) | All status | 2026-06-10 | Condition/disease: kidney transplant; Intervention/treatment: eculizumab | 16 |
| ClinicalTrials.gov (wide search) | All status | 2026-06-10 | Other terms: (eculizumab OR Soliris) AND (kidney OR renal) AND (transplant OR allograft) | 25 |
| WHO ICTRP | All source registration libraries | 2026-06-10 | eculizumab AND kidney transplant | 22 registration records, corresponding to 11 trials |
| WHO ICTRP | All source registration libraries | 2026-08-20 | eculizumab AND kidney transplant | 2 |
| WHO ICTRP | All source registration libraries | 2026-08-20 | Soliris AND kidney transplant | 11 |
| WHO ICTRP | All source registration libraries | 2026-08-20 | Soliris AND renal transplant | 0 |
| WHO ICTRP | All source registration libraries | 2026-08-20 | eculizumab AND antibody mediated rejection | 25 |
| WHO ICTRP | All source registration libraries | 2026-08-20 | eculizumab AND antibody-mediated rejection | 25 |
| Europe PMC | All records on the platform | 2026-06-10 | (eculizumab OR soliris) AND (kidney OR renal) AND (transplant* OR allograft* OR graft*) | 4,278 |
| OpenAlex | Platform relevance search | 2026-06-10 | eculizumab kidney kidney transplant allograft | 1,490; export the first 200 items |
| Crossref | Platform relevance search | 2026-06-10 | eculizumab kidney kidney transplant allograft | Supplementary discovery sources; export limit is the first 1,000 items |
| Semantic Scholar | The platform returns all records | 2026-06-10 | eculizumab kidney transplant | 604 |
| Citation tracking (OpenAlex and Web of Science) | Forward and backward citations of 8 core reports | 2026-06-10 | 10.1111/j.1600-6143.2011.03757.x；10.1111/ajt.13168；10.1111/ajt.15175；10.1111/ajt.15364；10.1111/ajt.15397；10.1016/j.transproceed.2017.12.015；10.3389/fimmu.2024.1465851；10.1016/j.trim.2025.102213 | 204 unique citation network records |
| ATC, ASN Kidney Week and ERA/NDT conference website | Access to meeting archives | 2026-06-10 | Search item by item: eculizumab kidney; eculizumab renal; eculizumab transplant; Soliris kidney; eculizumab antibody-mediated rejection; eculizumab AMR; eculizumab ABMR; eculizumab crossmatch; eculizumab desensitization; eculizumab ABO incompatible | 7 records with similar themes are subject to special verification |

**Supplementary Table S2. Studies excluded after full-text review, with reasons**

| **Literature (first author, year)** | **Title** | **DOI or other identifier** | **Reasons for exclusion** |
| --- | --- | --- | --- |
| Miura，2026 | Measurement of complement proteins may aid interpretation of pathophysiology in kidney transplant recipients with atypical hemolytic uremic syndrome. | 10.1007/s13730-026-01114-5 | Evaluated prevention or treatment of aHUS/TMA rather than AMR prophylaxis |
| Mirioglu，2026 | Thrombotic microangiopathy after kidney transplantation: diagnosis and management strategies. | 10.1093/ckj/sfag018 | Evaluated prevention or treatment of aHUS/TMA rather than AMR prophylaxis |
| Bertrand，2025 | Early Thrombotic Microangiopathy After ABO-Incompatible Living Donor Kidney Transplantation. | 10.1016/j.ekir.2024.12.031 | Evaluated prevention or treatment of aHUS/TMA rather than AMR prophylaxis |
| Kinoshita，2024 | Novel factors potentially initiating acute antibody-mediated rejection in pig kidney xenografts despite an efficient immunosuppressive regimen. | 10.1111/xen.12859 | Non-human allogeneic kidney transplantation; this study is a pig kidney xenotransplantation study. |
| Kanbay，2024 | The role of anticomplement therapy in the management of the kidney allograft. | 10.1111/ctr.15277 | Review/regulatory article, not original clinical AMR prevention research. |
| Yamamoto，2022 | Case report: Successful simultaneous heart-kidney transplantation across a positive complement-dependent cytotoxic crossmatch. | 10.3389/fneph.2022.1047217 | In cases of combined heart-kidney transplantation during the same period, the AMR preventive effect of kidney transplantation cannot be separated. |
| Li，2022 | De novo systemic atypical hemolytic uremic syndrome in an ABO-incompatible living kidney transplant recipient with a novel pathogenic CFHR1 gene mutation successfully treated with eculizumab: a case report. | 10.1007/s40620-022-01391-0 | Evaluated prevention or treatment of aHUS/TMA rather than AMR prophylaxis |
| Spasovski，2022 | Pharmacotherapeutic options for the prevention of kidney transplant rejection: the evidence to date. | 10.1080/14656566.2022.2102418 | Review, guideline, or other non-original research |
| Ishida，2021 | Desensitization Regimen Consisting of High-Dose Intravenous Immunoglobulin, Plasmapheresis, and Rituximab (an Anti-CD20 Antibody), Without Eculizumab and/or Bortezomib, in 41 Highly Sensitized Kidney Transplant Recipients. | 10.6002/ect.2021.0234 | No eculizumab intervention. |
| Stojanovic，2021 | Early relapse of atypical hemolytic uremic syndrome following ABO-incompatible living-related pediatric kidney re-transplant successfully treated with eculizumab. | 10.1007/s00467-021-05193-7 | Evaluated prevention or treatment of aHUS/TMA rather than AMR prophylaxis |
| Viklicky，2021 | Developments in immunosuppression. | 10.1097/mot.0000000000000844 | Review, guideline, or other non-original research |
| Bhalla，2020 | Complement-Based Therapy in the Management of Antibody-Mediated Rejection. | s1548-5595(19)30229-0 | Narrative review, no original clinical AMR prevention cohort. |
| Sendogan，2019 | Therapeutic Effect of Plasmapheresis and Intravenous Immunoglobulin in Recipients of Kidney Transplant With High Panel-reactive Antibody Levels: A Single-center Experience. | s0041-1345(18)31735-4 | The study intervention was plasma exchange and IVIG, with no eligible eculizumab prophylaxis group. |
| Böhmig，2018 | Novel Approaches to Block Complement. | 10.1097/tp.0000000000002267 | Review complement blocking article, no original clinical cohort. |
| Legendre，2017 | The role of complement inhibition in kidney transplantation. | 10.1093/bmb/ldx037 | Review article, no original clinical AMR prevention data. |
| Levi，2017 | Midterm Outcomes of 12 Renal Transplant Recipients Treated With Eculizumab to Prevent Atypical Hemolytic Syndrome Recurrence. | 10.1097/tp.0000000000001909 | Mainly for the prevention and treatment of aHUS/TMA recurrence, the preventive role of AMR is inseparable. |
| Lanfranco，2017 | Eculizumab for Thrombotic Microangiopathy Associated with Antibody-Mediated Rejection after ABO-Incompatible Kidney Transplantation. | 10.1155/2017/3197042 | Evaluated prevention or treatment of aHUS/TMA rather than AMR prophylaxis |
| Tatapudi，2017 | Pharmacologic Complement Inhibition in Clinical Transplantation. | 10.1007/s40472-017-0148-7 | Review of complement suppression in clinical transplantation, no original work can be included in the cohort. |
| Yamamoto，2017 | Efficacy of Eculizumab Therapy for Atypical Hemolytic Uremic Syndrome Recurrence and Antibody-Mediated Rejection Progress After Renal Transplantation With Preformed Donor-Specific Antibodies: Case Report. | s0041-1345(16)30768-0 | Mainly for the prevention and treatment of aHUS/TMA recurrence, the preventive role of AMR is inseparable. |
| Paterno，2016 | Successful Simultaneous Liver-Kidney Transplantation in the Presence of Multiple High-Titered Class I and II Antidonor HLA Antibodies. | e121 | In cases of combined liver-kidney transplantation during the same period, the AMR preventive effect of kidney transplantation cannot be separated. |
| Orandi，2016 | Splenic Irradiation for the Treatment of Severe Antibody-Mediated Rejection. | 10.1111/ajt.13882 | Evaluated treatment of established rejection or TMA rather than prophylaxis |
| Jordan，2016 | Progress in Desensitization of the Highly HLA Sensitized Patient. | s0041-1345(16)00129-9 | Review articles without isolated original clinical cohort or case data. |
| Eskandary，2016 | Complement inhibition as potential new therapy for antibody-mediated rejection. | 10.1111/tri.12706 | Review article, not original clinical research. |
| Johnson，2015 | Eculizumab use in kidney transplantation. | 10.1097/mot.0000000000000249 | Review article on eculizumab in kidney transplantation, no original clinical cohort. |
| Rostaing，2015 | Efficacy of immunoadsorption to reduce donor-specific alloantibodies in kidney-transplant candidates. | 10.6002/ect.mesot2014.o169 | The study is immunoadsorption desensitization, and there is no eligible eculizumab preventive intervention group. |
| Rostaing，2015 | Efficacy and safety of tandem hemodialysis and immunoadsorption to desensitize kidney transplant candidates. | PMID:25894148 | The study is hemodialysis combined with immunoadsorption desensitization, and there is no eligible eculizumab prevention group. |
| Kaabak，2014 | Eculizumab reverses the potentially fatal effects of kidney graft reperfusion injury. | 10.1111/petr.12206 | Eculizumab is used to rescue renal graft reperfusion injury that has occurred. The main problem is the prevention of IRI rather than AMR. |
| Legendre，2013 | Eculizumab in kidney transplantation. | 10.1016/j.trre.2013.04.002 | Review article on eculizumab in kidney transplantation, no original clinical cohort. |
| Barnett，2013 | The use of eculizumab in kidney transplantation. | 10.1111/ctr.12102 | Review article, no original clinical AMR prevention data. |
| Nester，2013 | Eculizumab in the treatment of atypical haemolytic uraemic syndrome and other complement-mediated renal diseases. | 10.1097/mop.0b013e32835df4a3 | Review aHUS and other complement-mediated nephropathy and briefly describe AMR prevention, no original separable cohort. |
| Stewart，2012 | Case report: Eculizumab rescue of severe accelerated antibody-mediated rejection after ABO-incompatible kidney transplant. | s0041-1345(12)00668-9 | Evaluated treatment of established rejection or TMA rather than prophylaxis |
| Pascual，2012 | Chronic renal allograft injury: early detection, accurate diagnosis and management. | s0955-470x(12)00033-x | A review of the diagnosis and treatment of chronic graft injury, not an original ECU-AMR prevention study. |
| Noone，2012 | Antibody mediated rejection associated with complement factor h-related protein 3/1 deficiency successfully treated with eculizumab. | 10.1111/j.1600-6143.2012.04124.x | Eculizumab is used to treat AMR that has already occurred, not to prevent it. |
| Morath，2012 | Recent developments in desensitization of crossmatch-positive kidney transplant recipients. | 10.1016/j.transproceed.2012.04.006 | Review articles without original clinical research data. |
| Blume，2012 | Antibody-mediated rejection: pathogenesis, prevention, treatment, and outcomes. | 10.1155/2012/201754 | Review of pathophysiology, prevention and treatment of AMR, no original clinical cohort. |
| Ruffoni，2012 | [Role of antibodies in kidney transplant]. | PMID:22388831 | Review/non-original study of antibodies in kidney transplantation, no separate ECU prophylaxis cohort. |
| Biglarnia，2011 | Prompt reversal of a severe complement activation by eculizumab in a patient undergoing intentional ABO-incompatible pancreas and kidney transplantation. | 10.1111/j.1432-2277.2011.01290.x | In cases of combined pancreas-kidney transplantation during the same period, the AMR preventive effect of kidney transplantation cannot be separated from the multi-organ situation. |
| van den Hoogen，2011 | Use of monoclonal antibodies in kidney transplantation. | 10.2217/imt.11.72 | Review of kidney transplantation monoclonal antibodies, no original ECU-AMR prevention cohort. |
| Marfo，2011 | Desensitization protocols and their outcome. | 10.2215/cjn.08140910 | This is a review article, not an original clinical AMR prevention study. |
| Stegall，2010 | Deciphering antibody-mediated rejection: new insights into mechanisms and treatment. | 10.1097/mot.0b013e3283342712 | A review of the mechanism and treatment of AMR, no original clinical cohort. |
| Khan，2024 | Thrombotic Microangiopathy in ABO-incompatible Kidney Transplant – Eculizumab to the Rescue | 10.4103/ijot.ijot_8_23 | Evaluated prevention or treatment of aHUS/TMA rather than AMR prophylaxis |
| Bababekov，2018 | Goal-directed Management of Coagulation: The Right Treatment, the Right Patient, the Right Time. | 10.1097/tp.0000000000002179 | The DOI/title corresponds to a review of perioperative coagulation management in liver transplantation and does not have an abstract; the original record is mixed with irrelevant AMR text, which is a contamination of the content of the title and is not a clinical issue in this study. |
| Castaneda，2022 | Thrombotic Microangiopathy Secondary to COVID-19 in a Recent ABO-Incompatible Kidney Transplant | 10.26502/acmcr.96550463 | Evaluated prevention or treatment of aHUS/TMA rather than AMR prophylaxis |
| Yabu，2020 | Should we use immunodominant DSA to measure response to therapy in antibody-mediated rejection? | 10.1097/tp.0000000000003146 | The study focused on DSA monitoring of patients with AMR treatment response and no eligible preventive intervention with eculizumab. |
| Rufino Hernandez，2011 | Desensitization and Induction Immunosuppressive Therapy in Highly HLA-Sensitized Patients Receiving Cadaveric Renal Allograft) | 10.5772/17406 | Book chapters/review materials, indissociable original clinical cohorts. |
| Rho，2025 | #1523 Two cases of hyperacute rejection after AB0 incompatible kidney transplantation – unexpected antibodies | 10.1093/ndt/gfaf116.1881 | There is no eligible prophylactic eculizumab intervention in cases of ABOi kidney transplant hyperacute rejection. |
| Moncada，2025 | #2594 Complement system evaluation in blood group incompatible living donor kidney transplantation | 10.1093/ndt/gfaf116.0820 | The study was a complement system evaluation of ABOi living donor kidney transplantation, and there was no eligible preventive intervention with eculizumab. |
| Thervet，2012 | Vaccination in solid-organ transplantation candidates: time for a benefit/risk assessment | 10.1093/ckj/sfs063 | Vaccination editorial/risk assessment without eculizumab AMR prevention intervention. |

**Supplementary Table S3. Linkage of multiple reports and cohort overlap among included studies**

One line per study or registered project. Among the 31 included reports, 30 can be classified into the following 14 items; the cohort source of the Brealey 2018 single-case pathology report cannot be confirmed and is presented as independent pathology background data. [33]

| **study or cohort** | **main report** | **Accompanying and follow-up reports** | **Report content** | **Cohort overlap** | **Outcome source used for synthesis** | **Approach to report linkage** | **References** |
| --- | --- | --- | --- | --- | --- | --- | --- |
| Stegall-positive cross-match living donor transplant cohort and extended follow-up | Stegall 2011 | Goh 2012; Long-term follow-up abstracts 2014; Bentall 2014; Cornell 2015; Dean 2017; Schinstock 2019 and its conference abstracts | Early AMR comparison, PLEX exposure, mechanistic and histological analysis, mid- and long-term graft and biopsy outcomes | The early 26 ECU recipients were part of the subsequent expansion cohort of 30 cases; the mechanism and conference reports were from the same cohort and expanded follow-up. | Stegall provides early comparisons; Cornell and Schinstock provide extended and long-term outcomes; companion reports supplement mechanism, pathology, or rescue therapy | Each outcome uses the largest available non-duplicate cohort at the corresponding time point. | [15,19–20,41,46–49] |
| Marks 2019 Living Donor Transplantation Randomized Trial | Marks 2019 | NCT01399593; Lefaucheur 2018 cross-trial post hoc analysis | Randomized comparison of treatment failure, central pathology AMR, graft and safety; C1q stratified pathology analysis | Registration records correspond to the same trial; Lefaucheur only included selected subgroups of the trial | Marks provides randomized comparison results; registration records supplement study design; Lefaucheur provides C1q stratified results | Registration records and post-hoc subgroup assignments merged with the main trial | [16–17,50] |
| Glotz 2019 Highly Sensitized Dead Donor Kidney Transplantation Trial | Glotz 2019 | NCT01567085; 2015 one-year follow-up summary; Lefaucheur 2018 cross-trial post hoc analysis | Single-arm AMR, PLEX, graft, safety, and C1q stratified pathological results | Registration, follow-up abstract and main article belong to the same 80-case trial; Lefaucheur used selected subgroups | Glotz main text is used for cohort and primary outcome; other reports supplementary design or descriptive pathology results | The 80-case cohort is counted only once; subgroups are not added to the full cohort | [16,18,51–52] |
| Chicago ABO Incompatible Transplant Program | West-Thielke 2018；Heagler 2022 | Campara 2018 infection safety summary | Multiple-dose regimen in 4 cases, infection outcomes in 4 cases, and single-dose regimen in 9 cases | West-Thielke and Campara are the same 4 cases; the overlap between 9 Heagler cases and 4 earlier cases cannot be ruled out | Four case reports provide multiple-dose regimens and outcomes; Campara supplements infection data; 9 case abstracts describe single-dose regimens | The efficacy and safety of 4 cases belong to the same cohort; due to unclear overlap, 4 cases and 9 cases were not totaled. | [6,21,36] |
| Heo 2024 High Titer ABO Incompatibility Case Series | Heo 2024 | ASN 2024 TH-PO818 Abstract | Intensive desensitization, pathology, graft function and infection results in 2 cases | The abstract and full text of the meeting are from the same two patients. | The peer-reviewed full text provides the main data, and the abstract supplements the reporting attribution. | The full text and abstract are merged into the same 2 case cohort. | [7,53] |
| Mitomo 2025 Peri-reperfusion Single ECU Case Series | Mitomo 2025 | No companion reports identified | Scheme, pathology and kidney function of 2 cases of high-titer ABO incompatible transplantation | No overlap with other cohorts found | Full text for all available results | Count once as 2 case series | [8] |
| Wan 2026 ABO Incompatibility Comparative Study | Wan 2026 | No errata, supplementary material, or author clarifications identified | Short-term rejection, kidney function and infection outcomes of ECU combined with DFPP and control | No overlap with other studies was found; there was a denominator conflict between 30 cases/group and 70 cases/group in the article | Denominators and results from different sources are presented separately. | Comparative effects were not calculated due to conflicting denominators. | [37] |
| Yoshikawa 2025 Two-dose ECU plus belatacept case series | Yoshikawa 2025 Conference Summary | No complete journal article | Protocol, short-term kidney function and infection in 3 cases of positive flow cross-match transplantation | It may be related to the Chicago co-center project, but the patient overlap cannot be confirmed | Results are presented as 3 cases in the abstract report | Not aggregated with other same-center cohorts | [22] |
| Okumi 2026 Mixed Indication Cases | Okumi 2026 | No companion reports identified | Complement blockade, pathology, and one-year outcomes in patients with positive crossmatch and suspected aHUS | Independent case; ECU used for both AMR prevention and suspected aHUS treatment | Provide treatment and outcome data for mixed indications | Not included in pure AMR prevention event rate | [23] |
| Shimmura 2017 high titer ABO incompatibility case | Shimmura 2017 Conference Summary | Withdrawn 2015 conference abstract | Single-case ECU induction, antibody removal, and long-term AMR outcomes | The same case was reported in 2015 and 2017 | 2017 follow-up abstract provides outcome information | The withdrawn 2015 conference abstract and the subsequent follow-up abstract describe the same case | [24,54] |
| NCT01106027 Positive cross-match deceased donor kidney transplantation study | ClinicalTrials.gov result record NCT01106027 | No full text of identified journals | Dosing regimen, subject flow, primary endpoints and adverse events of 2 patients | No overlap found with Stegall and its extended cohorts | Registration results provide sample, protocol and outcome information | The primary outcome was based on 1 completer, and the safety outcome was based on 2 treatment recipients. | [40] |
| NCT06453135 ABO incompatibility prevention registry protocol | ClinicalTrials.gov registration record NCT06453135 | No results reported yet | Planned enrollment, single ECU strategy, and 6-month primary endpoint | Independent registry protocol | Reflect studies that have not yet reported results | Uncountable patients or events | [34] |
| Milan ECU combined with obinutuzumab project | 2023 Extension Conference Summary | 2022 7 Cases Early Session Summary | Multimodal induction, historical comparison, AMR and infection in highly sensitized deceased donor kidney transplantation | The 7 cases reported in 2022 are likely to be part of the 10-case expansion cohort in 2023; the AMR reports are 2/7 and 0/10, which is a conflict | Denominators and outcomes are presented separately for both sources; the 2023 summary provides historical comparative context | Due to possible overlap in cohorts, 7 cases and 10 cases were not totaled; conflicting AMR results were not combined. | [38–39] |
| ChiCTR2500114806 high titer ABO incompatible registration scheme | ChiCTR2500114806 registration record | No results reported yet | Planned peri-transplant ECU protocol, inclusion criteria, and predetermined endpoints | Independent registry protocol | Reflect studies that have not yet reported results | Uncountable patients or events | [35] |

**Supplementary Table S4. Risk of bias and funding disclosures for included studies**

Funding and conflicts of interest are descriptive information and are not considered independent bias areas in RoB 2, ROBINS-I or JBI tools. NR or failure to report does not equate to confirmation that no funding or conflict of interest exists.

| **Study** | **Rating object/design** | **Tool** | **Key domain/item-level judgments** | **Overall judgment** | **Funding** | **Conflicts of interest** | **References** |
| --- | --- | --- | --- | --- | --- | --- | --- |
| Stegall study and extended follow-up | AMR historical comparison | ROBINS-I V2 (November 2025 draft) | Confounding is a critical risk; selection into the study and outcome measurement are serious risks; other areas are low to moderate risk. | Very high risk of bias | Alexion is the study sponsor or provides research support. | The lead author reports a research contract or previous research funding with Alexion. | [15,19–20,41] |
| Marks 2019 | Prespecified 9-week composite treatment failure and central pathology Banff grade II–III AMR | RoB 2 | There are some concerns about the randomization process and outcome measurement; deviations from the intended intervention, missing outcomes, and selective reporting were considered low risk. | Some concerns | Alexion funded research and medical writing support. | One author was an employee of Alexion at the time of the study; several authors report consulting, research funding, or travel affiliations. | [17] |
| Glotz 2019 | 9-week AMR and primary safety outcomes | JBI Critical Appraisal Checklist for Cohort Studies (items applicable to a single-arm cohort) | Enrollment, exposure and follow-up are relatively clear; there is no concurrent control and the indications for rescue treatment are not fully reported. | Not overall graded; see key entries | Funded by Alexion. | One author is an employee of Alexion; several authors report consulting, speakership, travel, or institutional trial support. | [18] |
| Chicago ABO Incompatible Items | A case series of 4 multiple-dose and 9 single-dose cases | JBI Critical Appraisal Checklist for Case Series | Inclusion criteria, consecutive enrollment, complete inclusion, and reporting of results were unclear. | Not overall graded; see key entries | Accessible case reports and conference abstracts did not indicate specific funding. | The accessible sources do not provide a complete statement of conflicts of interest. | [6,21,36] |
| Heo 2024 | A case series of 2 cases of ABO incompatibility | JBI Critical Appraisal Checklist for Case Series | The case selection is strong and the joint intervention is intensive; the outcome follow-up is relatively complete. | Not overall graded; see key entries | Yonsei University Internal Science Research Fund DUCR00095. | The authors declare no commercial or financial conflicts of interest; one author serves on the editorial board of the journal. | [7] |
| Mitomo 2025 | A case series of 2 cases of ABO incompatibility | JBI Critical Appraisal Checklist for Case Series | The case selection is strong and the joint intervention is intensive; the outcome follow-up is relatively complete. | Not overall graded; see key entries | The original report had no external funding. | The original article reports no competing interests. | [8] |
| Wan 2026 | non-randomized comparative study | ROBINS-I V2 (November 2025 draft) | Grouping patterns, confounding controls, and rejection definitions were missing, and there were internal sample size conflicts. | Very high risk of bias | Not reported. | Not reported. | [37] |
| Yoshikawa 2025 | 3 case conference case series | JBI Critical Appraisal Checklist for Case Series | There was insufficient information in the meeting abstract, and continuous enrollment, complete inclusion, and AMR determination could not be evaluated. | Not overall graded; see key entries | No funding was reported in the conference abstract. | No conflicts of interest were reported in the conference abstract. | [22] |
| Okumi 2026 | Mixed indication single case | JBI Critical Appraisal Checklist for Case Reports | The single case report is relatively complete, but the indications for AMR prevention and aHUS cannot be separated. | Not overall graded; see key entries | There is no special funding for the original report. | Okumi reports consulting and lecturing fees from Alexion. | [23] |
| Shimmura 2017 | Single case of high titer ABO incompatibility | JBI Critical Appraisal Checklist for Case Reports | The conference single case information was limited and the co-interventions were intensive. | Not overall graded; see key entries | No funding was reported in the conference abstract. | No conflicts of interest were reported in the conference abstract. | [24] |
| NCT01106027 | Registration results of 2 patients receiving treatment | JBI Critical Appraisal Checklist for Case Series | Only 2 cases were registered, and different denominators were used for different outcomes. | Not overall graded; see key entries | Registration records list study sponsor; no independent journal funding statement. | Registration results do not provide an author conflict of interest statement. | [40] |
| NCT06453135 | Registry-only protocol with no posted results | Not applicable (no reported results) | No clinical results. | Not rated | Registered programs do not report dedicated funding. | No resulting paper is available for verification. | [34] |
| Milan 2022/2023 | Historical control comparison of conflicting AMR results | ROBINS-I V2 (November 2025 draft) | Historical comparisons are uncorrected, cohort versions overlap and AMR results conflict. | Very high risk of bias | No funding was reported in the conference abstract. | A full statement of conflicts of interest is not provided in the meeting abstract. | [38–39] |
| ChiCTR2500114806 | Registry-only protocol with no posted results | Not applicable (no reported results) | No clinical results. | Not rated | Registered programs do not report dedicated funding. | No resulting paper is available for verification. | [35] |

**Supplementary Table S5. Sensitivity analyses using alternative AMR definitions in the Marks randomized trial**

Both RR and RD were calculated from the number of reported events; RD was expressed in percentage points, and its 95% CI used the Newcombe hybrid-score method without continuity correction. Central review following Grade I–III analysis and provision of clinical data does not replace the prespecified centrally blinded Grade II/III primary definitions.

| **Pathological interpretation methods** | **AMR level** | **level of analysis** | **ending** | **ECU events/n** | **Control event/n** | **RR（95% CI）** | **RD, percentage point (95% CI)** | **References** |
| --- | --- | --- | --- | --- | --- | --- | --- | --- |
| Prespecified central pathology assessment; blinded to treatment group; no clinical data provided | Grade II/III | Main analysis | composite treatment failure | 5/51 | 7/51 | 0.71 (0.24 to 2.10) | −3.92 (−17.14 to 9.22) | [17] |
| Prespecified central pathology assessment; blinded to treatment group; no clinical data provided | Grade II/III | Main analysis | AMR component events | 5/51 | 5/51 | 1.00 (0.31 to 3.25) | 0.00 (−12.47 to 12.47) | [17] |
| Central pathology reinterpretation; blinded to treatment group; provision of clinical data | Grade II/III | Post hoc central review | composite treatment failure | 6/51 | 11/51 | 0.55 (0.22 to 1.36) | −9.80 (−24.28 to 4.94) | [17] |
| Central pathology reinterpretation; blinded to treatment group; provision of clinical data | Grade II/III | Post hoc central review | AMR component events | 6/51 | 9/51 | 0.67 (0.26 to 1.74) | −5.88 (−19.96 to 8.26) | [17] |
| Local pathology interpretation; clinical data available | Grade II/III | Prespecified sensitivity analysis | composite treatment failure | 7/51 | 15/51 | 0.47 (0.21 to 1.05) | −15.69 (−30.93 to 0.39) | [17] |
| Local pathology interpretation; clinical data available | Grade II/III | Prespecified sensitivity analysis | AMR component events | 7/51 | 12/51 | 0.58 (0.25 to 1.36) | −9.80 (−24.73 to 5.51) | [17] |
| Central pathology reinterpretation; blinded to treatment group; provision of clinical data | Grade I/II/III | post hoc sensitivity analysis | composite treatment failure | 6/51 | 15/51 | 0.40 (0.17 to 0.95) | −17.65 (−32.61 to −1.85) | [17] |
| Central pathology reinterpretation; blinded to treatment group; provision of clinical data | Grade I/II/III | post hoc sensitivity analysis | AMR component events | 6/51 | 13/51 | 0.46 (0.19 to 1.12) | −13.73 (−28.50 to 1.57) | [17] |
| Local pathology interpretation; clinical data available | Grade I/II/III | post hoc sensitivity analysis | composite treatment failure | 10/51 | 21/51 | 0.48 (0.25 to 0.91) | −21.57 (−37.70 to −3.70) | [17] |
| Local pathology interpretation; clinical data available | Grade I/II/III | post hoc sensitivity analysis | AMR component events | 10/51 | 18/51 | 0.56 (0.28 to 1.08) | −15.69 (−31.87 to 1.67) | [17] |

**Supplementary Table S6. Infection and other safety outcomes**

One row per study arm with safety data. NR means not reported in the original report, which is different from zero events that are clearly reported; the definition of serious infection or clinically important infection is inconsistent between studies. The 7 early cases in Milan are likely to be included in the 10 expanded cases, and the overlap between the 4 early cases in Chicago and the 9 late cases in Chicago cannot be confirmed, so the total sample size was not calculated. Studies without available safety data are not included in this table.

| **research and research arms** | **n** | **Follow-up** | **serious adverse events** | **Serious/Clinically Important Infections** | **meningococcal infection** | **CMV** | **BK virus** | **EBV** | **other infections** | **References** |
| --- | --- | --- | --- | --- | --- | --- | --- | --- | --- | --- |
| Marks 2019; ECU combined with standard treatment after desensitization before transplantation | 51 | Ending specific; see individual event cells | 43/51 (until the last study visit) | 32/51 (until the last research visit) | 0/51 (Full research process) | NR | NR | NR | NR | [17] |
| Marks 2019; Standard treatment after pre-transplant desensitization | 51 | Ending specific; see individual event cells | 48/51 (until the last study visit) | 25/51 (until the last research visit) | 0/51 (Full research process) | NR | NR | NR | NR | [17] |
| Glotz 2019; ECU single arm | 80 | Ending specific; see individual event cells | 70/80 (36 months) | 65/80 (36 months) (study-defined clinically important infection) | NR | NR | NR | NR | NR | [18] |
| Chicago ABO Incompatible Project; Chicago Early Multiple Dose ECU Cohort | 4 | Ending specific; see individual event cells | NR | NR | 0/4 (during the reporting follow-up period) | 1/4 (during the reporting follow-up period) | NR | NR | Any infection 2/4 (reported during follow-up period) | [6,21,36] |
| Chicago ABO Incompatible Program; Chicago Late Single Dose ECU Cohort | 9 | Ending specific; see individual event cells | NR | NR | NR | NR | NR | NR | Any infection 6/9 (during the follow-up period listed in the table) | [6,21,36] |
| Heo 2024; Adding short-course ECU to refractory ABO-incompatible desensitization | 2 | Ending specific; see individual event cells | NR | NR | NR | NR | 1/2 (3 months) | NR | Other major infections 0/2 (12 months) | [7] |
| Wan 2026; control protocol in report | 30 | Ending specific; see individual event cells | NR | NR | NR | NR | NR | NR | Any infection 4/30 (3 months) | [37] |
| Wan 2026; ECU combined with DFPP test plan | 30 | Ending specific; see individual event cells | NR | NR | NR | NR | NR | NR | Any infection 1/30 (3 months) | [37] |
| Yoshikawa 2025; two-dose ECU plus belatacept regimen | 3 | Ending specific; see individual event cells | NR | NR | NR | 0/3 (6 months) | 0/3 (6 months) | NR | Any infection 0/3 (6 months) | [22] |
| Okumi 2026; Mixed indication regimen of ECU combined with IVIG, PE and rituximab | 1 | Ending specific; see individual event cells | NR | NR | NR | NR | NR | NR | NR | [23] |
| Shimmura 2017; Addition of ECU after intensive DFPP, PEX, IVIG, and anti-CD20 desensitization | 1 | Ending specific; see individual event cells | NR | NR | NR | NR | NR | NR | NR | [24] |
| NCT01106027; Addition of ECU to standard care and one planned pre-transplant plasma exchange | 2 | Ending specific; see individual event cells | 1/2 (1 year) | NR | NR | NR | NR | NR | Pneumonia 1/2 (1 year) | [40] |
| Milan 2022/2023; Early ECU combined with obinutuzumab multimodal induction | 7 | Ending specific; see individual event cells | NR | NR | NR | 4/7 (median follow-up 13 months) | 0/7 (median follow-up 13 months) | 1/7 (median follow-up 13 months) | NR | [38–39] |
| Milan 2022/2023; Extended ECU combined with obinutuzumab multimodal induction | 10 | Ending specific; see individual event cells | NR | 0/10 (median follow-up 16 months) (life-threatening infection) | NR | NR | NR | NR | NR | [38–39] |
| Milan 2022/2023; matched rituximab historical controls | 20 | Ending specific; see individual event cells | NR | 2/20 (median follow-up 16 months) (life-threatening infection) | NR | NR | NR | NR | NR | [38–39] |
